# A Simple Rule for Dendritic Spine and Axonal Bouton Formation Can Account for Cortical Reorganization after Focal Retinal Lesions

**DOI:** 10.1371/journal.pcbi.1003259

**Published:** 2013-10-10

**Authors:** Markus Butz, Arjen van Ooyen

**Affiliations:** 1 Simulation Lab Neuroscience - Bernstein Facility for Simulation and Database Technology, Institute for Advanced Simulation, Jülich Aachen Research Alliance, Forschungszentrum Jülich, Jülich, Germany; 2 VU University Amsterdam, Amsterdam, The Netherlands; Université Paris Descartes, Centre National de la Recherche Scientifique, France

## Abstract

Lasting alterations in sensory input trigger massive structural and functional adaptations in cortical networks. The principles governing these experience-dependent changes are, however, poorly understood. Here, we examine whether a simple rule based on the neurons' need for homeostasis in electrical activity may serve as driving force for cortical reorganization. According to this rule, a neuron creates new spines and boutons when its level of electrical activity is below a homeostatic set-point and decreases the number of spines and boutons when its activity exceeds this set-point. In addition, neurons need a minimum level of activity to form spines and boutons. Spine and bouton formation depends solely on the neuron's own activity level, and synapses are formed by merging spines and boutons independently of activity. Using a novel computational model, we show that this simple growth rule produces neuron and network changes as observed in the visual cortex after focal retinal lesions. In the model, as in the cortex, the turnover of dendritic spines was increased strongest in the center of the lesion projection zone, while axonal boutons displayed a marked overshoot followed by pruning. Moreover, the decrease in external input was compensated for by the formation of new horizontal connections, which caused a retinotopic remapping. Homeostatic regulation may provide a unifying framework for understanding cortical reorganization, including network repair in degenerative diseases or following focal stroke.

## Author Summary

The adult brain is less hard-wired than traditionally thought. About ten percent of synapses in the mature visual cortex is continually replaced by new ones (structural plasticity). This percentage greatly increases after lasting changes in visual input. Due to the topographically organized nerve connections from the retina in the eye to the primary visual cortex in the brain, a small circumscribed lesion in the retina leads to a defined area in the cortex that is deprived of input. Recent experimental studies have revealed that axonal sprouting and dendritic spine turnover are massively increased in and around the cortical area that is deprived of input. However, the driving forces for this structural plasticity remain unclear. Using a novel computational model, we examine whether the need for activity homeostasis of individual neurons may drive cortical reorganization after lasting changes in input activity. We show that homeostatic growth rules indeed give rise to structural and functional reorganization of neuronal networks similar to the cortical reorganization observed experimentally. Understanding the principles of structural plasticity may eventually lead to novel treatment strategies for stimulating functional reorganization after brain damage and neurodegeneration.

## Introduction

The mature brain is not as hard-wired as traditionally thought. Long-term in vivo imaging has revealed that dendritic spines appear and disappear frequently, accompanied by synapse formation and elimination [1]. Spine and synapse formation and elimination are induced by learning [2]–[4] and are associated with long-term memory storage [5]–[7]. Similarly, peripheral lesions, which permanently alter input to cortical areas, trigger extensive spine formation and elimination [8]–[11]. Likewise, large-scale axonal sprouting and pruning in cortical areas are associated with focal retinal lesions [12], [13], whisker trimming [8], and digit or limb amputation [14], [15]. Axonal and dendritic arborizations are profusely intertwined [16], so a neuron can already access a large pool of neurons by just extending its dendritic spines or slightly changing the length of its neurites (axons or dendrites).

Despite the relevance of structural changes for cortical adaptations after alterations in sensory input [1], [9], [10], the driving forces behind structural plasticity remain elusive. Particularly, it is unknown whether neuronal structural changes may be induced primarily by the neuron's own activity level or critically depend on the activity level of other neurons as well, as in associative forms of synaptic plasticity such as STDP. We therefore investigated whether the need of neurons to maintain their average electrical activity at a particular level (homeostatic regulation) could account for the changes in neuronal morphology and the functional rewiring of cortical circuitry observed in the primary visual cortex after focal retinal lesions [10], [13].

Electrical activity controls the growth of axons [17]–[21] and dendrites [22]–[26], and the formation of spines [11], [27]–[30] and boutons [13]. Alterations in dendritic membrane potential can induce very localized structural changes that seem to follow Hebbian synaptic plasticity rules [31]. After lesions, however, when the activity of the whole neuron is altered, the soma may innitiate compensatory structural changes of the entire neuronal morphology. The way in which the neuron's electrical activity influences neurite outgrowth and synapse numbers suggests that neurons try to maintain their level of electrical activity at a particular set-point (homeostasis) [32]–[36]. When electrical activity is below the homeostatic set-point and above a certain mimimal level, neurons extend their dendrites towards sources of activity [37], [38] and enhance spine formation [1]. When electrical activity is too high or very low (below a required minimal level), neurons arrest neurite outgrowth and eliminate spines [11], [39]–[43].

What global network dynamics may arise from such local activity-dependent changes in neuronal morphology? Changes in neuronal morphology affect synaptic connectivity, altering neuronal and network activity, which in turn changes neuronal morphology. The consequences of these reciprocal interactions are hard to predict from experimental studies alone. Computational modelling is required to help elucidate these complex dynamics. Most computational models [44], [45], however, consider network structure as fixed, with plasticity merely arising from changes in connection strengths (synaptic plasticity). To explore what structural changes in neurons and network circuitry may emerge after a loss of input, we used a novel computational model, called the *structural plasticity model* [36], [46], [47], in which a neuron creates new spines and boutons when its activity level is within an optimal range, and delete spines and boutons when activity is outside this range. We found that the dendritic and axonal dynamics in the model after a loss of external input were remarkably similar to the formation and deletion of dendritic spines in mice [10] and the changes in axonal boutons in monkeys [13] after focal retinal lesions. The model generated, as an emergent property of the local activity-dependent rules for structural plasticity, an axonal overshoot and subsequent pruning of horizontal connections that re-occupy deafferented neurons and so cause a retinotopic remapping. Our results demonstrate that functional reorganization in terms of cortical remapping can result from structural rewiring of cortical circuitry alone and does not necessarily require associative forms of synaptic plasticity such as STDP [48]. The model generates a variety of testable predictions with respect to cortical circuit rewiring following changes in sensory input.

## Model

### The whole model at a glance

**Figure 1:**
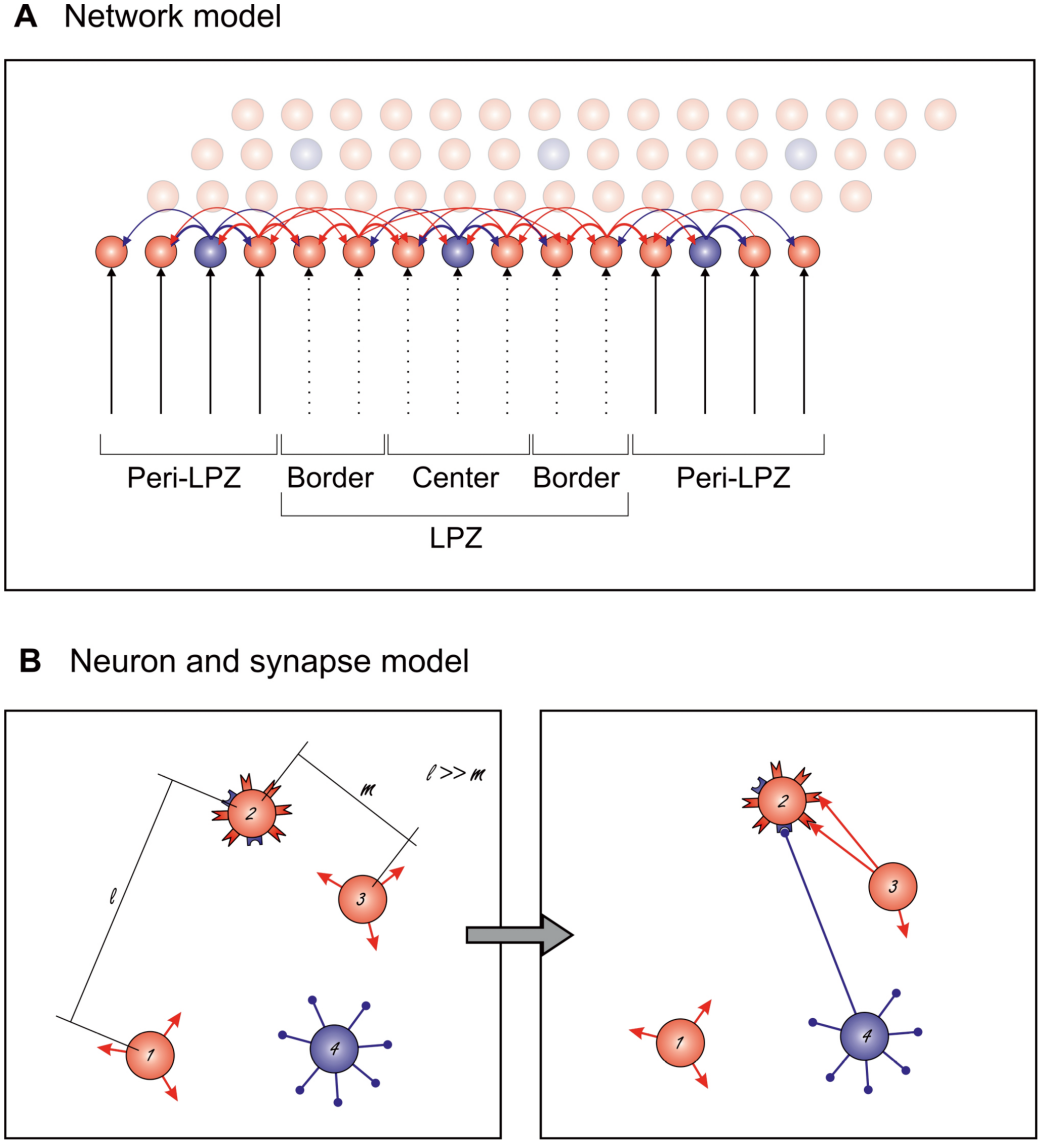
Schematic representation of our *structural plasticity model*. The model is organized as a two-dimensional recurrent network resembling the canonical cortical microcircuit. A) The cortical network consists of (excitatory) pyramidal cells (red) and inhibitory interneurons (blue). Neurons receive input from other neurons in the cortex and external input from the eye via the thalamus. A circumscribed loss of external input defines the lesion projection zone, which can be further subdivided into the center and the border. The peri-LPZ consists of neurons that surround the LPZ. Neurons are interconnected by directed synapses that have a fixed strength. The linewidth indicates the number of connections between pairs of neurons. B) Synapses consist of re-combinable axonal (‘plugs’) and dendritic elements (‘sockets’), enabling synapse formation, deletion and rewiring. Synaptic elements are either excitatory (red) or inhibitory (blue). Complementary axonal and dendritic elements merge to form synapses. The probability that a synapse is formed between two neurons depends on the number of elements that each neuron has and on the Euclidean distance between the neurons. That is, synapse formation from neuron 3 to 2 with distance 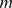 is more likely than from neuron 1 to 2 with distance 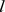, given that 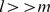 and neurons 1 and 3 have equal numbers of vacant axonal elements.

As shown in mice [10] and monkeys [13], a circumscribed loss of input to cortical networks triggers massive structural changes in neuronal morphology. We used our *structural plasticity model* (Fig. 1) to explore the impact of a loss of input (referred to as ‘lesion’) on network rewiring caused by activity-dependent changes in axonal and dendritic morphology. A circumscribed area in the model, corresponding to the lesion projection zone (LPZ) in the animal experiment, is deprived of external input. The LPZ can be further subdivided into a center and a border region. Whereas the border region still receives some input via horizontal connections from outside the LPZ, the neurons in the center region are almost completely deprived of input. In accordance with the experimental literature, the area with intact external input directly surrounding the LPZ is defined as the peri-LPZ. In order to be able to predict what changes occur at the network level as a result of activity-dependent changes at the cellular level, the model is detailed enough to include measurable physiological variables such as membrane potential, spiking, intracellular calcium concentration and numbers of dendritic spines and axonal boutons, yet computationally simple enough to characterize network changes such as cortical rewiring and functional remapping.

From a theoretical point of view, the probability that two neurons form new synapses or break existing ones depends on, in addition to the distance between the neurons, the number of synaptic contact possibilities that each neuron has, i.e. the number of axonal boutons and dendritic spines. In principle, changes in spine and bouton numbers can be caused directly by changes in the numerical densities of spines and boutons or indirectly by neurite outgrowth and retraction. Since we are interested in the effective change in synaptic contact possibilities, we abstracted away from the detailed neuronal morphology by using one-compartment neurons that carry sets of synaptic elements representing axonal boutons (axonal elements), excitatory dendritic spines (excitatory dendritic elements) and inhibitory postsynaptic densities (inhibitory dendritic elements). Excitatory and inhibitory neurons form exclusively excitatory or inhibitory axonal elements, respectively, but can express both excitatory and inhibitory dendritic elements. Synaptic elements can develop independently of potential contact partners [49], [50] and are highly selective in connecting to matching synaptic counterparts [51], which motivated our idea of representing axonal and dendritic elements as ‘plugs’ and ‘sockets’ (cf. Fig. 1). Synapses are formed by merging corresponding synaptic elements or are deleted when synaptic elements are lost. Thus, activity-dependent changes in the number of synaptic elements alter synaptic connectivity.

The execution of the model procedes as follows. We continuously determine for each neuron its electrical activity on a millisecond timescale 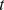. Electrical activity causes influx of calcium. Calcium concentration also decays, so the neuron's intracellular calcium concentration effectively represents the time-averaged level of its electrical activity. The intracellular calcium concentration then drives continuous changes in the number of synaptic elements per neuron [23]. Structural changes, i.e. changes in the number of synaptic elements, are much slower than changes in electrical activity, so reorganization of network connectivity and electrical activity dynamics take place on different time scales and do not directly interfere with each other. Because formation and deletion of synaptic elements occur continuously over time (reflecting an implicit growth process) but the formation of synapses (by merging synaptic elements) and breaking of synapses are singular events, we update connectivity at discrete time steps. Since changes in synaptic elements are slow and the algorithm for updating connectivity is computationally costly, we do the update in connectivity only every 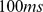. The structural dynamics are slow enough to be comparable to the experimentally observed morphological changes in the visual cortex of rodents after focal retinal lesion over a time span of 72 days. However, we did not unnecessarily slow down structural plasticity in the model to the point that the entire simulation time would sum up to 72 days in milliseconds.

In every connectivity update, we determine for each neuron how many vacant synaptic elements are available for synapse formation, and if the number of synaptic elements decreased, which of its synaptic elements, either vacant or bound in a synapse, should be removed. In every connectivity update, deletion of bound synaptic elements immediately causes the breaking of synapses. Vacant synaptic elements (i.e. not bound in a synapse) spontaneously decay with a certain time constant. For synapse formation, we distribute vacant synaptic elements on complementary elements in a random, distance-dependent manner, i.e. excitatory axonal elements bind to excitatory dendritic elements, and inhibitory axonal elements connect to inhibitory dendritic elements. Synapse formation is more likely between adjacent than between remote neurons. This scheme is repeated for every update in connectivity.

An important feature of the *structural plasticity model* is that after the breaking of a synapse, the complementary synaptic element remains, i.e. the axonal element if the dendritic element was deleted or the dendritic element if the axonal element was deleted. The vacant synaptic element is available for synapse formation again, so synaptic connections can rewire.

### Neuron model for generating electrical activity

The *structural plasticity model* used to model cortical deafferentation resembles the canonical cortical microcircuit [52] consisting of (excitatory) pyramidal cells and inhibitory, GABA-ergic interneurons. The mircocircuit is represented by a two dimensional recurrent network of 400 neurons, of which 80% are excitatory and 20% inhibitory [53]. In the model, excitatory and inhibitory neurons differ only in the sign of synaptic transmission; all other parameters are the same. Excitatory neurons were placed with a spatial variance of 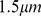 on a 20×16 grid with a distance between two grid points of 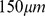. More precisely, the x,y-coordinates of each neuron were derived from a normal distribution (with the chosen spatial variance as standard deviation) that was centered at an individual grid point. For the 80 inhibitory neurons we defined a second 10×8 grid positioned in such a way that the inhibitory neurons become equally distributed among the excitatory ones; the precise x,y coordinates were determined as was done for the excitatory neurons. Given that neurons in the adult cortex of rodents [54] are capable of rewiring their axonal branches over a couple of hundred micrometers, the expected distance between neurons of 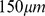 in the model is a plausible choice.

Neurons generate electrical activity. To describe neuronal firing, we used the Izhikevich model [55], a biophysical neuron model that provides a good trade-off between physiological realism and computational cost. In this model, the membrane potential 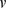 of a neuron is computed by two differential equations:
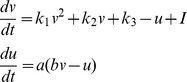
(1)where 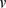 is in 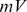, time 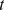 is in 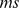, 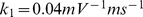, 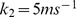, 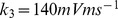, 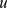 is a membrane recovery variable and 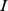 represents the input to the neuron. Each time the membrane potential exceeds 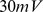 (i.e. fires a spike), 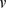 and 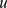 are reset to their resting values:
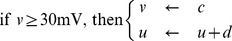
(2)


Parameters were set to 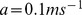, 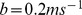, 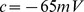, 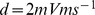. Each neuron receives input 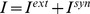 that comprises synaptic input 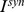 from other neurons in the network (horizontal input from adjacent and distant cortical neurons) and external input 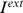 (vertical input from the eye via the thalamus). Neurons interchange electrical signals on a millisecond timescale without a synaptic delay. Synaptic input consists of the incoming action potentials from the presynaptic neuron low-pass filtered by an exponential filter function 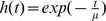 with decay constant 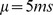. Network connectivity 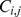 is defined as the number of synapses from neuron 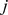 to 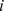. If a synapse exists, it has a fixed strength of 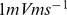. Neurons are either excitatory or inhibitory. Indices refer to excitatory neurons if 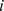 or 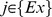 and to inhibitory neurons if 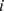 or 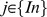. As in the original version of the neuron model [55], we defined the external input 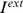 as a noisy current (white noise) with mean 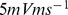 and standard deviation 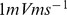. In [56], the total thalamocortical input to individual neurons in the primary visual cortex was estimated experimentally to be about 5 mV. However, this study did not report on the frequency of this input. The frequency may roughly be estimated by the number of thalamocortical synapses impinging on an individual neuron in the primary visual cortex and by the firing frequency of the thalamocortical projection neurons. Synapse numbers have been estimated to be about one hundred (cf. chapter 8, page 323 in [57]), while the firing frequency is about 10 Hz for each projection neuron [58]. If one hundred projection neurons fire with an expected firing rate of 10 Hz, it is perhaps reasonable to assume that 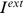 with input amplitude 5 mV is delivered on average at every millisecond.

### Electrical activity triggers changes in neuronal morphology

Although it is not exactly known how neuronal morphology in the visual cortex changes after loss of input due to focal retinal lesions, existing experimental data, e.g. from tissue culture, strongly support that changes in neuronal morphology are activity-dependent and that a certain level of activity is optimal for axonal and dendritic outgrowth and spine and bouton formation [33] (reviewed in [32]; cf. also Introduction). The intracellular calcium concentration 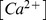 can be used as an indicator of a neuron's average electrical activity [59]–[61]. In the model, 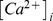 increases by a fixed amount 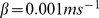 every time neuron 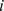 fires, and otherwise decreases exponentially to zero [61] with decay time 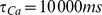. With these values of 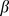 and 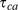 the intracellular calcium concentration represents the time-averaged electrical activity of a neuron and the concentration decay is of the same magnitude as measured experimentally [62].
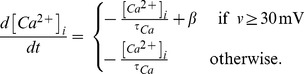
(3)


Through changes in the level of intracellular calcium, electrical activity modulates actin and tubulin polymerization and therefore neurite outgrowth [33]. If the average electrical activity of a neuron exceeds some maximum level, it will withdraw dendritic spines [63], [64] and retract neurite branches [65], which may reduce connectivity and hence activity. If activity becomes lower, the neuron will generate vacant synaptic elements [66], increasing connectivity and activity. However, if activity comes below some minimum level, outgrowth and synapse formation will be halted [10], [42], [43], [67]. These findings are further supported in a study by Richards et al. [30] showing that reducing electrical activity in hippocampal slice cultures increases the number of spine head protrusions, whereas this increase does not occur with a complete block of activity. Earlier studies [27], [28] are in line with these results. Thus, we conclude that homeostatic regulation in combination with a minimal level of electrical activity required for outgrowth may serve as a guiding principle for the formation of synaptic elements, i.e. for neurite outgrowth [17], [33] and spine and bouton formation [68], [69].

### Update in synaptic elements represents changes in neuronal morphology

Synapses consist of merged pairs of synaptic elements, i.e. axonal boutons and dendritic spines. Changes in the number of synaptic elements can be brought about by outgrowth or retraction of neurites, or by changes in the numerical density of these elements on neurites. In the model, we abstract over these two possibilities and consider merely the number of synaptic elements rather than the detailed morphology of the neuron. We define 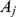 as the number of axonal elements per neuron, representing the overall number of axonal boutons. Likewise, we define 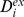 and 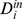 as the number of excitatory and inhibitory dendritic elements, respectively, representing excitatory dendritic spines and inhibitory postsynaptic densities. Dendritic elements can be excitatory or inhibitory regardless of the type of the hosting neuron, whereas all axonal elements of a neuron are either excitatory or inhibitory. In the numerical integration, 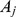, 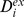 and 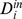 are treated as continuous variables, but when synaptic elements are deleted or used for synapse formation, the values of 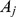, 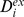 and 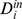 are rounded off to their smallest integer values.

**Figure 2:**
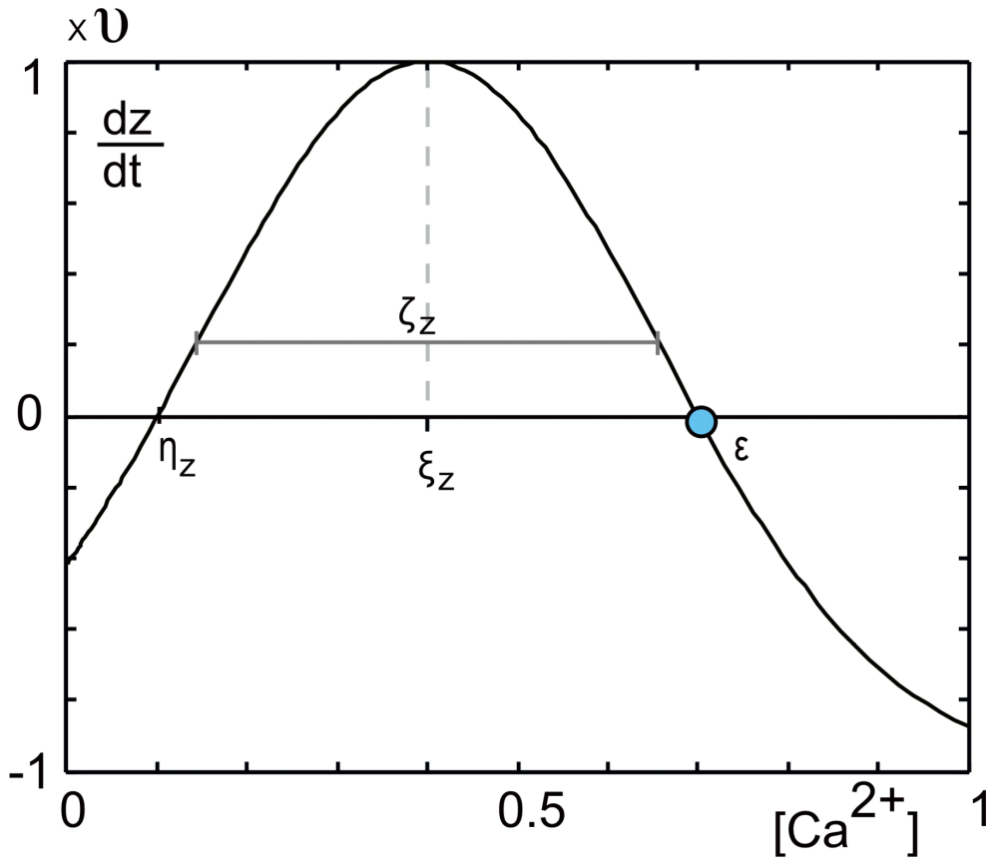
The graph depicts the principal shape of the growth curve, giving the change 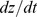 in each type of synaptic elements 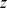 as a function of the calcium concentration. Parameter 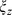 is the center and 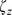 is the width of the Gaussian curve. Parameters 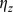 and 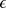 are the intersections with the x-axis.

We postulate Gaussian-shaped growth curves for the activity-dependent formation and deletion of every type of synaptic element, i.e. excitatory and inhibitory axonal elements 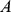, excitatory dendritic elements 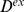 and inhibitory dendritic elements 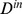:
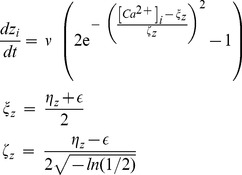
(4)where 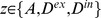 is the continuous number of synaptic elements. By choosing 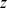, we obtain the growth curve of a particular type of synaptic element (Fig. 2). Eq. 4 applies to the total number of synaptic elements of the respective type regardless of whether synaptic elements are bound in a synapse or unbound.

Growth curves are bounded to 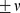, where 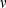 is the maximum speed of increase or decrease in the number of synaptic elements, with 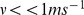. We used 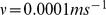, which is small enough to represent the slow time scale of growth yet large enough not to slow down the simulations unnecessarily. Growth curves are further determined by a low 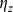 and a high set-point 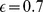, at which points there are no activity-dependent changes in the number of the respective type of synaptic element. Parameter 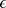 is a stable fixed point to which the system will converge when 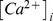 of all neurons 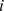 is higher than 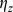. The value of 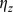 depends on the type of synaptic element (cf. Results section for the choice of 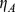 and 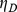), whereas 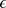 is the same for all types. The center and width of the Gaussian-shaped growth curve (Fig. 2) are given by 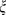 and 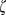, respectively. Synaptic elements that are not bound in a synapse (vacant elements) decay with time constant 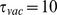 connectivity updates.
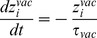
(5)


For reasons of simplicity and because of lack of detailed experimental constraints, we used the same type of growth rules for both axonal and dendritic elements. We also used identical growth rules for excitatory and inhibitory synaptic elements.

### Update in network connectivity due to synapse deletion

Every 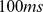 we perform an update in connectivity based on the activity-dependent continuous change in 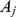, 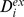 and 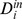. The continuous changes determine how many discrete synaptic elements on neuron 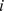 have to be deleted. For example, if neuron 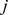 had previously 100 axonal elements bound in 100 outgoing synapses stored in 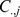 (with ‘.’ indicating the entire column of the connectivity matrix) and 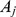 decreased to e.g. 95.32 due to Eq. 4, 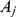 is rounded off to 95 and consequently neuron 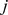 has to delete 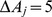 outgoing synapses at the next update in connectivity. For the update in connectivity, the algorithmic procedure then determines which connections to postsynaptic neurons are to be reduced. This is done by listing all 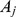 individual axonal elements per neuron 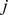 with the index of its postsynaptic target neuron 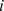 and randomly selecting 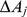 synaptic elements from this list for deletion. Vacant elements are included in this list and can be chosen for deletion as well. All synaptic elements have an equal chance to be deleted, regardless of whether they are vacant or bound in a synapse. The removal of a bound synaptic element immediately causes the breaking of the synapse, i.e. reducing the respective 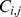 by one (or more if more than one element bound to a dendritic element on postsynaptic neuron 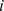 was chosen for deletion). Note that with breaking of synapses, the total number of dendritic elements of the postsynaptic neuron 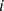 remains unchanged, but the number of vacant dendritic elements of neuron 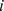 increases. The same procedure is done for all types of elements 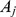, 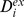 and 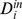 in every update in connectivity.

The above is the description of the algorithmic procedure implemented. Given that a number of synaptic elements 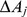, 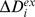 and 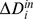 needs to be deleted, the expected loss of synapses 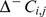 between any pairs of neurons 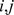 can be predicted analytically. For excitatory 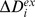 and inhibitory dendritic elements 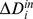 the change can be predicted as follows:
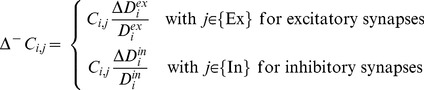
(6)with 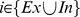.

Likewise, the expected loss of outgoing synapses caused by deletion of axonal elements of neuron 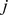 with either 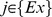 or 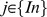 is
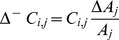
(7)If 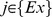, excitatory synapses will break, whereas if 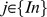 inhibitory synapses will break. In both cases, the postsynaptic neurons 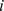 affected by synapse loss can be excitatory or inhibitory 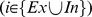.

### Update in network connectivity due to synapse formation

A neuron can undergo synapse formation when it has gained vacant synaptic elements. For each type of element, the number of vacant synaptic elements 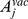, 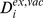 and 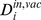 on neuron 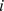 or 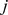 is the difference between the total number and the number of bound synaptic elements, with the continuous variables 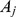, 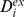, 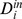 being rounded off to their smallest integer values (as described in “Update in synaptic elements due to synapse deletion”). The number of bound synaptic elements is equal to the number of incoming synapses (for bound dendritic elements) or outgoing synapses (for bound axonal elements) of a neuron. Thus,
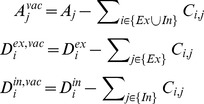
(8)


Initially, the numbers of bound and vacant synaptic elements are zero. Vacant synaptic elements may come from an increase in synaptic elements due to the activity-dependent growth rules (Eq. 4) or from the previous breaking of synapses formed earlier (Eq. 6–7). For synapse formation, all vacant synaptic elements from all neurons are randomly and simultaneously assigned to a complementary synaptic element, i.e. excitatory axonal elements to excitatory dendritic elements and inhibitory axonal elements to inhibitory dendritic elements. If there are more synaptic elements of a particular type than there are matching counterparts, some elements will remain vacant. Whether or not assigned pairs of complementary synaptic elements actually form synapses depends on the Euclidean distance between the neurons. A two-dimensional Gaussian kernel 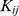 with width 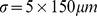 (where 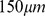 is the distance between two grid points) determines the distance-dependent likelihood for synapse formation between any pair of neurons:
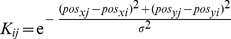
(9)with 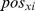 the x-coordinate and 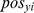 the y-coordinate of the location of postsynaptic neuron 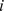, and 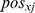 and 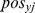 the coordinates of presynaptic neuron 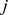 in 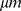.

The expected number of synapses to be formed from neuron 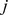 to 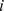 can be approximated on the basis of the number of vacant synaptic elements and the distance-dependent likelihood 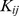:
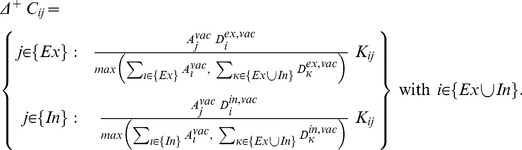
(10)


Eq. 10 provides, rather than a description of the algorithmic procedure implemented, an analytical approximation of the expected increase in synapse numbers for each update in connectivity. The denominator of Eq. 10 gives the maximum of the total number of vacant axonal elements and the total number of vacant dendritic elements in the entire network. The numerator is the product of the number of vacant axonal elements of presynaptic neuron 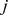 and the number of vacant dendritic elements of postsynaptic neuron 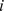. Eq. 10 can be interpreted as follows: Given that there are, for example, in total more vacant dendritic elements in the network than vacant axonal elements, the vacant axonal elements from neuron 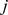 are proportionally distributed over all postsynaptic neurons offering dendritic elements. In this example, we would expect (ignoring for the moment the distance-dependent part of Eq. 10) that postsynaptic neuron 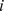 will connect proportionally to as many axonal elements of presynaptic neuron 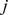 as the number of vacant dendritic elements that postsynaptic neuron 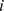 offers, namely 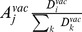. Hence, the allocation of vacant elements, and thereby the formation of new synapses, is more likely when a neuron has more vacant elements. The distribution of vacant synaptic elements, in combination with the neurons' distance to other neurons, determines the chance that a neuron connects to other neurons.

Note that for pairs of distant neurons, the expected number of synapses to be formed could be much below one because of a very low 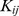. The expected number considerably increases when the number of vacant synaptic elements becomes higher. Such an increase in expected synapses can be interpreted as resulting from neurite outgrowth (not explicitly represented in the model). Initially, synapses between distant neurons are unlikely, but over time, when more vacant synaptic elements are produced, synapses between distant neurons will also become established. For every simulation run, networks start with zero connectivity and develop connectivity over time in a self-organizing process according to the procedure described above. Synapse formation and reorganization of network connectivity ceases when electrical activities of each neuron is close to the desired homeostatic set-point 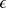.

### Defining a loss of external input as a model for focal retinal lesions

A rectangular lesion of the retina caused by photo-coagulation as described in [10] leads to a circumscribed deafferentation of the primary visual cortex—the lesion projection zone (LPZ, Fig. 1). The LPZ is modelled by depriving a central square of about 49 excitatory and 9 inhibitory neurons (numbers can slightly vary between simulations, since neurons are placed with some jitter on the two-dimensional plane, whereas the size and position of the square is fixed) of external input, i.e. by permanently setting 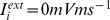. Before the lesion, all model neurons are at the high set-point of electrical activity, at which they do not considerably change their synaptic connectivity (‘mature’ network; cf. section “Electrical activity triggers changes in neuronal morphology”). This equilibrium state is usually obtained after 5000 connectivity updates, but to be absolutely sure that the network in all simulations reached an equilibrium, we applied lesions only after 8000 connectivity updates. After the lesion, simulations are then continued for another 5000 connectivity updates. In total, each simulation thus undergoes 13000 connectivity updates. In the time period after the lesion, we distinguished an early phase (the first 1000 connectivity updates after the lesion), a middle phase (2000–3000 connectivity updates) and a late phase (4000–5000 connectivity updates). The structural dynamics occuring over 5000 connectivity updates are comparable to the morphological changes observed experimentally over 72 days [10], although in the model the speed of structural dynamics is faster than in reality in order not to prolong the simulation unnecessarily. We compared all measurements to an unlesioned network (‘control’) after the same number of connectivity updates. Mann-Whitney U test or ANOVA was used with a Bonferroni post-hoc test, as described in the text. 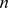 is the number of cells for each condition. All error bars denote standard deviation (SD) unless otherwise stated.

## Results

**Figure 3:**
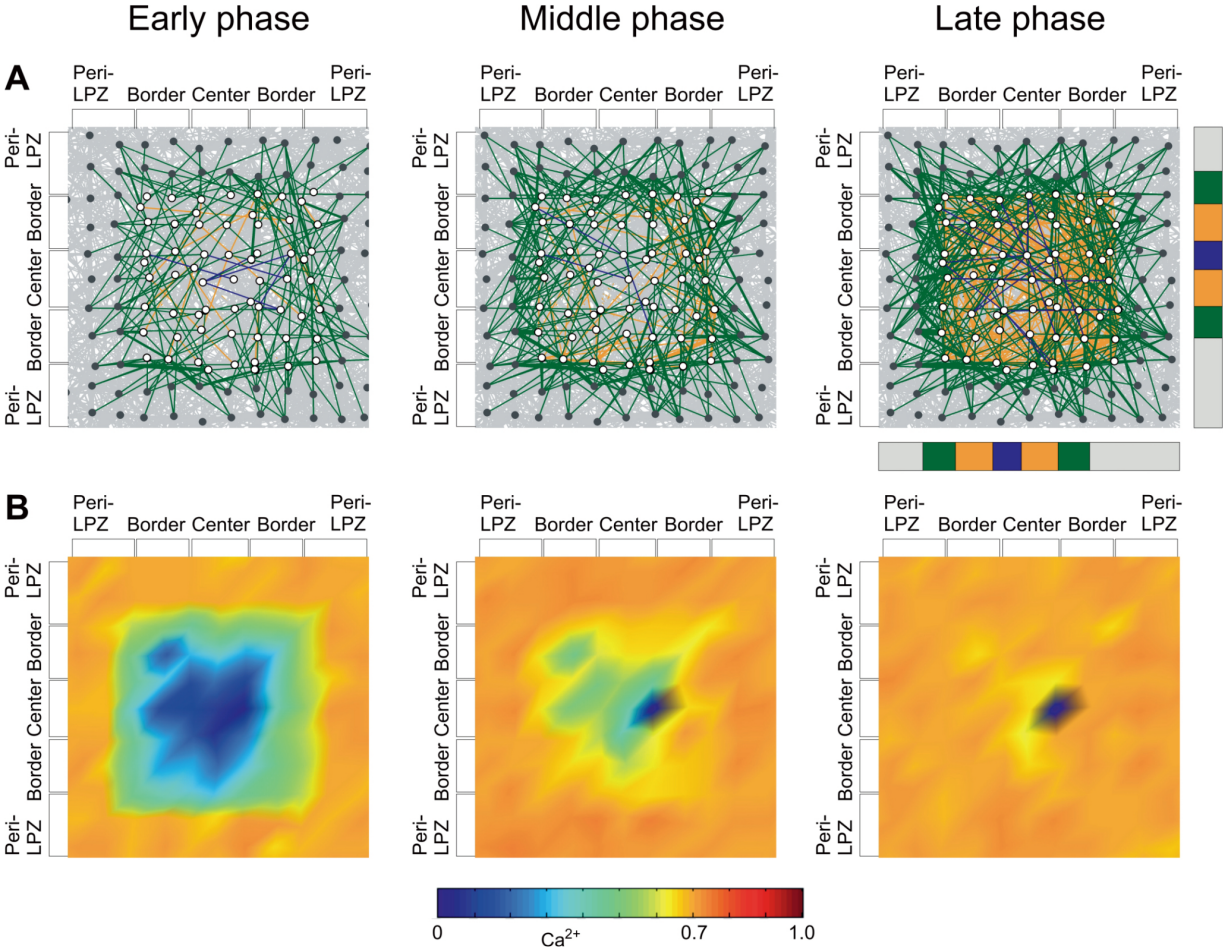
Network rewiring in the model leads to a sequential increase in electrical activity from the border to the center of the LPZ. A) Color-labeled are those synaptic connections that are newly formed after the lesion and impinge on neurons anywhere in the LPZ. Pre-existing and other connections are in gray, covering almost the entire background. Connections originating from neurons in the peri-LPZ are labeled green, from the border of the LPZ yellow and from the center blue. In the early phase, new connections are mainly formed from the peri-LPZ to the border of the LPZ. Due to fluctuations in activity, transient connections may also occur in the border or the center but usually disappear again later. In the middle phase, connections are formed from the border to the center, and finally in the late phase connections are added that originate from the center. B) In the early phase, new connections raise the activity in the border of the LPZ and subsequently in the center. Depending on the size of the LPZ, neurons in the center may fail to recover. Activities of individual neurons are interpolated to show the spatial distribution of activity in the LPZ and peri-LPZ. A) and B) are close-ups of the combined area of the LPZ and peri-LPZ. The relative size of the LPZ (border: orange; center: blue) and the peri-LPZ (green) to the entire network (gray) is indicated by the vertical and horizontal bars next to the upper right panel. Total number of neurons (excitatory and inhibitory): LPZ: 62 neurons; peri-LPZ: 71 neurons.

We observed different types of network reorganization after focal loss of input depending on the activity-dependent growth rules that we used. If dendritic elements start forming at lower activity levels than axonal elements (e.g. 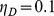 and 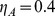), the increase in axonal and dendritic element numbers gives rise to network repair after a permanent loss of input. The network repair resembled the closure of a wound that is healing from the rim to the center. First, new axonal elements formed synaptic connections from the peri-LPZ to the border of the LPZ (Fig. 3a). Subsequently, axonal elements from the border of the LPZ established synapses with dendritic elements on neurons in the center. The increase in connectivity led to a sequential recovery of neurons from the border to the center of the LPZ, raising the neurons' calcium concentration and electrical activity to the high set-point 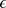 again (Fig. 3b).

**Figure 4:**
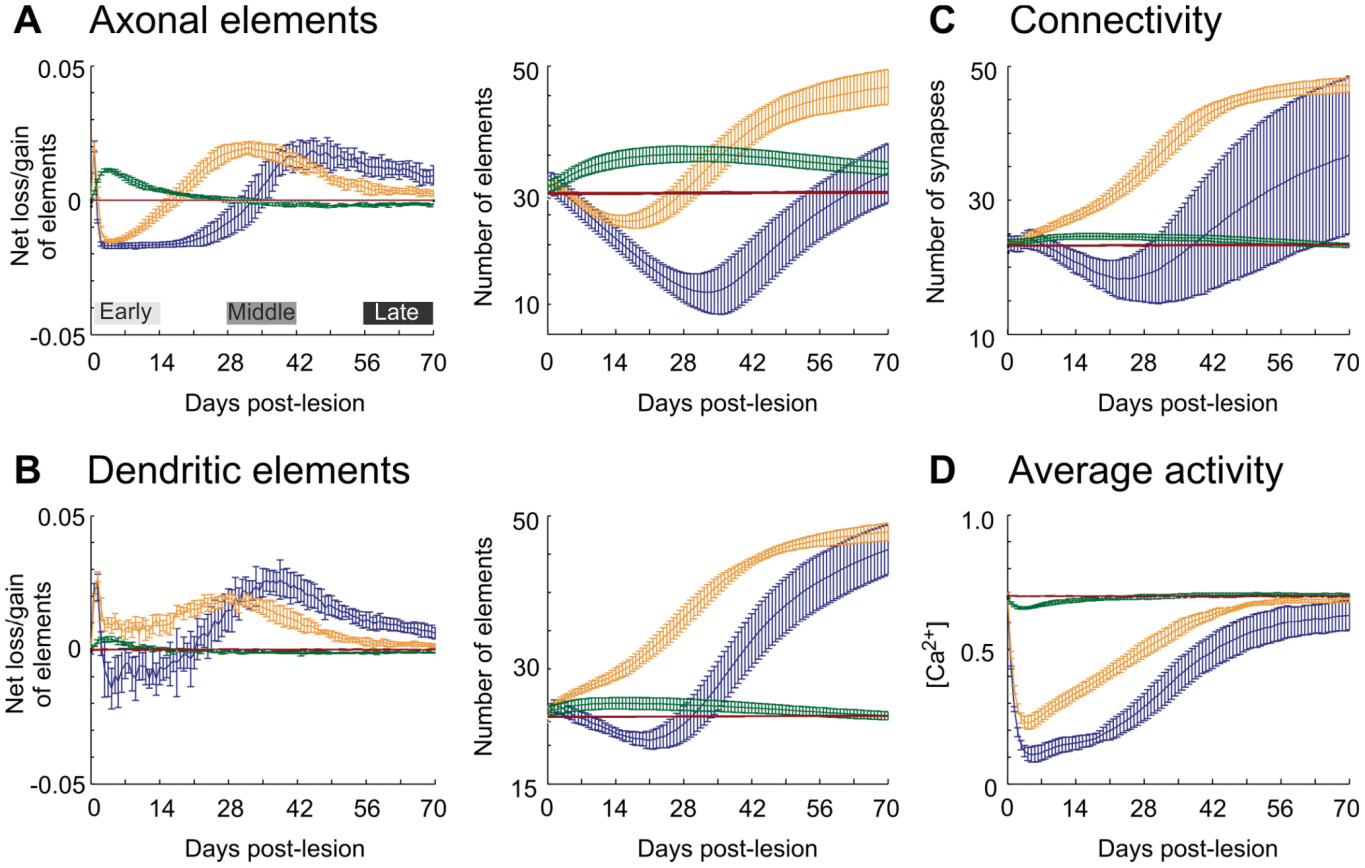
Local growth rules give rise to a change in the number of axonal and dendritic elements, which is crucial for network repair. Net changes are measured as loss or gain in A) axonal and B) dendritic elements on excitatory neurons between two time steps divided by the initial number of synaptic elements before the lesion. Also shown are the average number of synaptic elements per excitatory neuron. C) Total number of excitatory synapses impinging on excitatory neurons in the peri-LPZ, in the border and in the center of the LPZ. D) Increase in average electrical activity (represented by calcium concentration) over time of all neurons in the peri-LPZ, in the border and in the center of the LPZ. All measurements for the peri-LPZ are labeled green, for the border orange, for the center blue and for controls brown. All measurements are averaged over 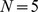 runs; error bars: SD. Bars in (A) indicate the early, middle and late phase after lesion.

Initially after the lesion, neurons in the peri-LPZ increase their number of axonal elements (Fig. 4). Dendritic elements are mostly formed in the border of the LPZ. The kernel function (Eq. 9) makes synapse formation more likely between adjacent neurons, thus supporting synapse formation between neurons in the peri-LPZ and the border of the LPZ. At the same time, axonal elements in the LPZ degenerate, causing the breaking of synapses in the LPZ and leaving behind vacant dendritic elements. Vacant synaptic elements can immediately be used for renewed synapse formation, but also decay over time when unbound in synapes (Eq. 5). The spontaneous decay of vacant dendritic elements and the formation of new dendritic elements lead to a substantial replacement of dendritic elements, particularly in the center of the LPZ, because most axonal elements from the peri-LPZ do not reach into the center. During the middle phase after the lesion, axonal elements are also provided by neurons in the border of the LPZ. With axonal elements now in reach, vacant dendritic elements on center neurons have a chance of connecting to axonal elements on border neurons. At the same time, pruning of unused axonal elements in the peri-LPZ sets in. During the late phase after the lesion, even neurons in the center may produce axonal elements and form synapses. The sequential formation of synapses repairs the network in the sense that most neurons return to their initial activity level.

**Figure 5:**
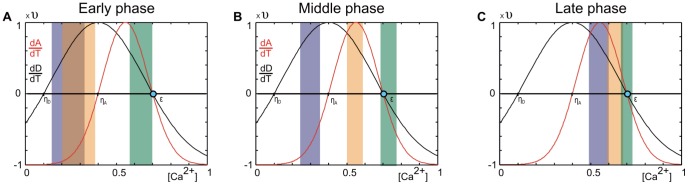
Differences in electrical activity in the LPZ and peri-LPZ account for network repair. For growth rules with low 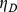 and high 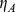 (here 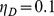 and 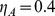), cooperative effects emerge between the peri-LPZ and LPZ that lead to a significant increase in activity in the center and the border of the LPZ. During the early phase (A), activities and calcium concentrations in the LPZ are higher than 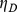 but lower than 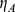, leading to dendritic element formation and reduction of axonal elements. In the middle phase (B), activities in the border region of the LPZ now exceed 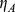 and cells in the border form axonal elements, too. In the late phase (C), all activities have returned close to the set-point 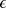. Growth curves for axonal elements are drawn in red and for dendritic elements in black. The cyan spot on the x-axis indicates the set-point 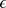. The colored bars schematically indicate the range of calcium concentration in the peri-LPZ (green), the border of the LPZ (orange), and the center of the LPZ (blue). Colored bars have transparent fillings to show overlaps.

The course of network repair is the result of reciprocal interactions between changes in activity, number of synaptic elements and connectivity. The process is essentially triggered by an initial difference in activity between the inside and outside of the LPZ (Fig. 5). Since neurons at the border of the LPZ are in an optimal activity range for dendritic element formation while neurons in the peri-LPZ are in an optimal range for axonal element formation, the chance of synapse formation is highest from peri-LPZ neurons to neurons in the border region of the LPZ. The minimum activity for axonal elements to grow is 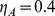, which is higher than the activity in the LPZ early after the lesion. Once the activity in the border of the LPZ has exceeded 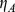, neurons in the border will also provide axonal elements. As soon as neuronal activities in the center of the LPZ exceed 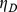, they form vacant dendritic elements that can be occupied by axonal elements from the border region of the LPZ. Finally, neurons in the center increase their activity and may also form axonal elements if their activity, too, exceeds 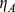.

Remarkably, without a neuron actually ‘knowing’ the activity levels of other neurons, the fact that neurons are subsequently at an optimal activity level for axonal and dendritic element formation leads to a very specific reorganization of network connectivity. That is, synapses are formed preferentially from neurons of higher activity (first in the peri-LPZ and later in the border of the LPZ) towards neurons of lower activity (first in the border of the LPZ and later in the center of the LPZ), even though the merging of axonal and dendritic elements itself is random and independent of electrical activity. The result of this sequential synapse formation is that neuronal activity is raised from the border towards the center of the LPZ. In the best case, all neurons in the LPZ are able to restore their activity level to the high set-point (cf. Eq. 4). Note that the sequential growth process is not imposed by the kernel function (Eq. 9) because principally different courses of reorganization can emerge from the same kernel when different growth rules are used (cf. Section “Aberrant network reorganization”).

However, neurons in the center are at risk of failing to recover if they are not able to form enough new synapses. This is especially the case when the size of the LPZ is too large (cf. “Aberrant network reorganization”). In this situation, the surplus of axonal elements is not sufficient to fulfil the demands of all the neurons deprived of input. Consequently, the support chain of axons, sequentially forming new synapses and increasing the activity of more central neurons, breaks down at some point, so network repair will not be complete. Network repair may also fail for other combinations of axonal and dendritic growth rules, as we discuss in section “Aberrant network reorganization”. Because 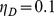 and 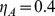 lead to network recovery, we compare the results obtained with these values with the experimental data.

### Comparison with experimental data

**Figure 6:**
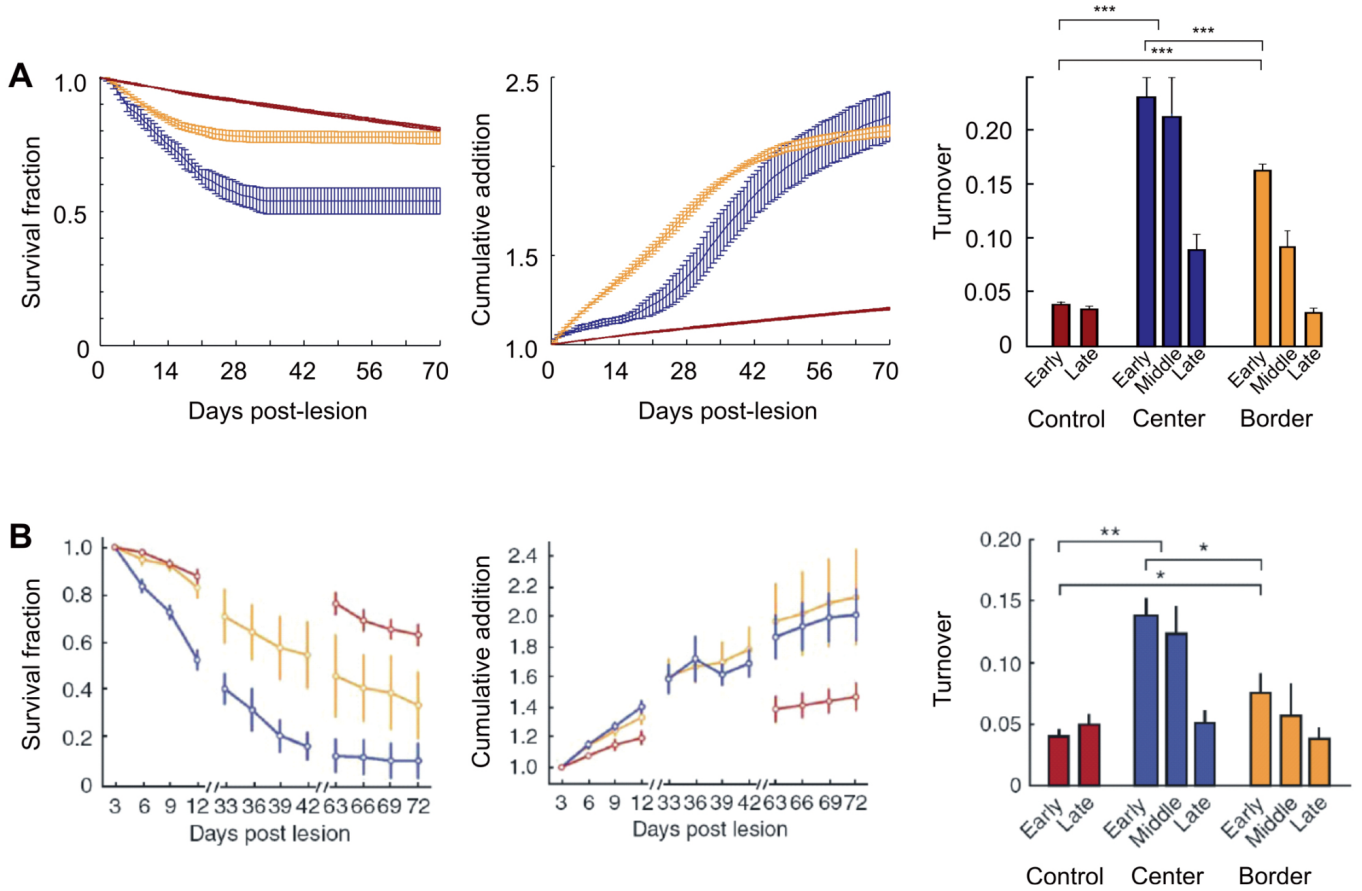
The dynamic changes in dendritic elements in the *structural plasticity model* show remarkable similarities to the spine dynamics in the visual cortex of mice after focal retinal lesions. The figure shows the survival fraction rates, accumulative addition rates and turnover rates of dendritic elements and dendritic spines on excitatory neurons A) in the model and B) in the experiment (experimental data in B reprinted by permission from Macmillan Publishers Ltd: Nature Neuroscience: Keck T et al. (2008) Massive restructuring of neuronal circuits during functional reorganization of adult visual cortex. Nat Neurosci 11:1162–1167, copyright 2008). Error bars in A) SD and in B) (experimental data:) SEM with center of LPZ: 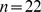 cells; border of LPZ: 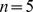 cells; control: 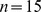 cells. Color code: center of the LPZ: blue; border of the LPZ: orange; control: brown.

Comparison with the experimental data revealed that growth rules with low 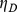 and high 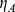 produced dynamics of axonal and dendritic elements that are remarkably similar to the axonal bouton [13] and dendritic spine changes [10] observed in the viusal cortex after a focal retinal lesion. In agreement with the experimentally observed dendritic spine dynamics, our model produced the highest turnover rate in dendritic elements in the center of the LPZ (Fig. 6), which was significantly higher than the turnover in the border region 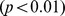, in the peri-LPZ 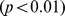 and in controls 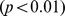. Turnover rate is defined as the sum of lost and gained dendritic elements/spines over a certain time period divided by twice the initial amount at the beginning of this period. In both the experiment and the model, also the border region showed an increased turnover rate compared to that in controls (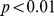; ANOVA with Bonferroni correction for multiple tests). Furthermore, strong similarities were found in survival fraction rate and cumulative addition rate between dendritic elements in the model and dendritic spines in the experiment [10]. Whereas the survival fraction rate indicates how many of the dendritic elements/spines before lesion are still present after lesion, the cumulative addition rate measures how many new spines compared to the initial number have been formed meanwhile. Interestingly, the survival fraction rates in both the experiment and the model showed a significant difference between the center and border of the LPZ (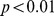 from post-lesion day 4 onwards in the model; Mann-Whitney U test), whereas the cumulative addition rates in the center and border region did not differ significantly (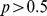 from day 45 onwards in the model). On the basis of our model results, we suggest the following explanation: The low activity level in the center of the LPZ leads to a stronger decrease in dendritic elements in the center as compared to the border and consequently to a lower survival fraction. While activity is the determining factor for dendritic element survival in the model —and possibly also for dendritic spines in the experiment—it is not the limiting factor for the cumulative addition of dendritic elements. In the model, the number of vacant axonal elements available for synapse formation and in reach for postsynaptic neurons is decisive for the cumulative addition rate of dendritic elements. Dendritic elements that did not bind to an axonal element quickly decayed and therefore limited the cumulative addition rates. Consequently, additional axonal boutons—in the visual cortex most likely provided by sprouting axonal branches [12], [13]—stabilize dendritic spines and thereby increase cumulative addition rates of dendritic spines. It remains unclear, however, what could determine the total gain in dendritic spines in the experiment. While there is a clear increase in spine density after a loss of input to the visual cortex caused by juvenile or adult monocular deprivation [9], the gain of total spine density after focal retinal lesions seems limited [10]. It is likely that if plasticity in synaptic strength was included in the model, fewer synaptic connections would be sufficient to enable neurons to return to the high set-point of electrical activity.

**Figure 7:**
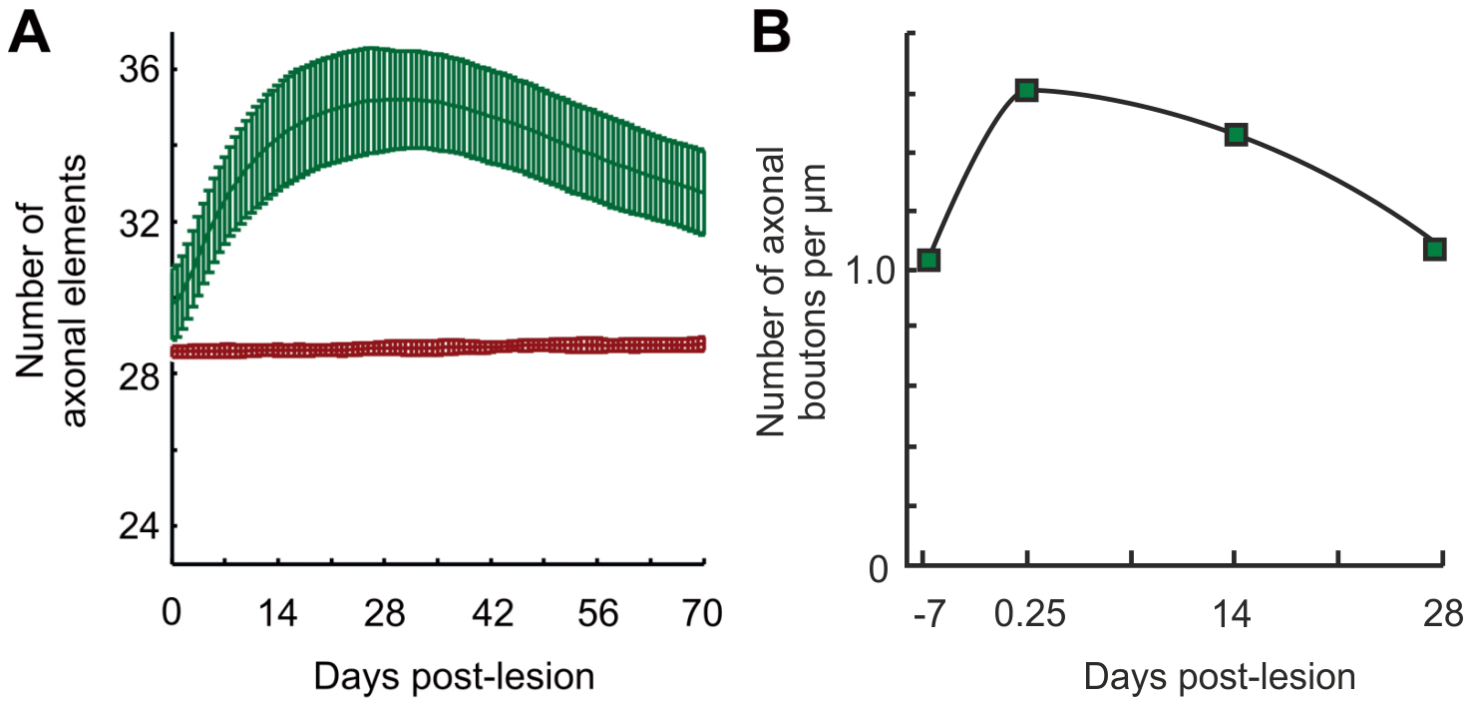
The model shows a significant overshoot in axonal elements on excitatory neurons over time following a lesion, as compared with the control situation without a lesion. A) The overshoot in the model is caused by axonal elements from neurons in the peri-LPZ (green line; brown: control). All measurements are averaged over 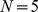 runs; error bars: SD. B) Although the available experimental data is very limited, there are two examples from an anterograde tracer study (labeling neurons in the peri-LPZ and measuring windows in the LPZ) in monkeys after focal retinal lesions that show a clear overshoot in axonal bouton densities of excitatory neurons. Green squares indicate points of measurement. Modified from [13].

Similarities between model and experiment were also observed with respect to axonal dynamics. The model produces an overshoot and subsequent pruning in number of axonal elements after the loss of input (Fig. 7). In the late phase after the lesion, the number of axonal elements remains significantly elevated compared to controls (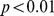, Mann-Whitney U test). In the model, the surplus of axonal elements is the source for additional horizontal connections from the peri-LPZ into the LPZ. The overshoot is caused by an early phase of decreased activity in the peri-LPZ followed by a middle phase of activity higher than the set-point 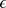 (cf. Fig. 5). Unfortunately, there is only limited experimental data on axonal growth into the LPZ after focal retinal lesions. The first examples were provided by [12], [70]. More recently, fine structure imaging of sprouting axonal branches and proliferating boutons indeed revealed an overproduction and subsequent pruning of axons and boutons [13]. The presence of axonal overshoot in the model strongly suggests that the dynamics in the model and the experiment are comparable. In conclusion, compensatory network rewiring as observed after lesions may be accounted for by local activity-dependent changes in individual neurons and the subsequent emerging cooperative effects between neurons inside and outside the LPZ.

### Functional implications of structural changes

**Figure 8:**
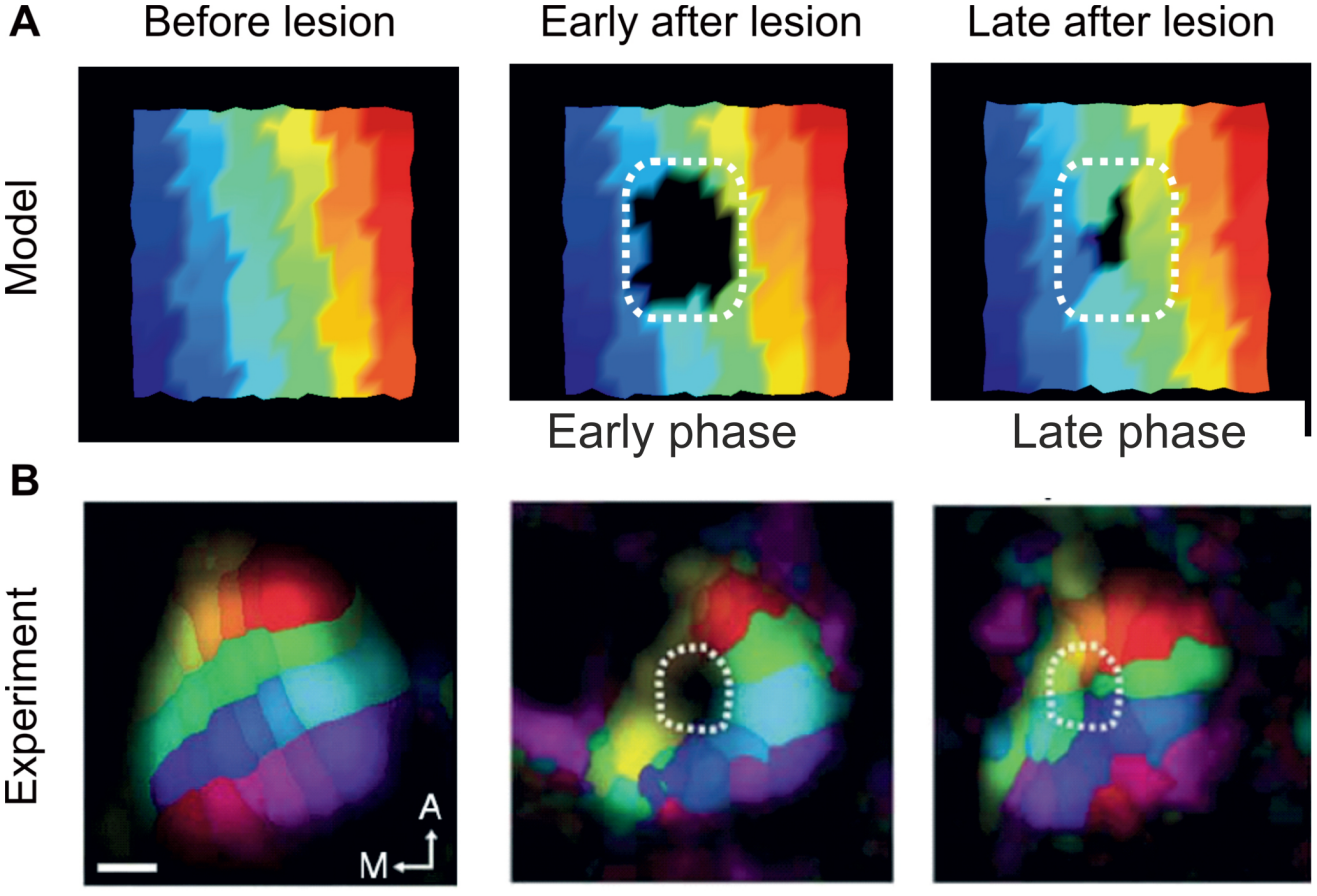
Network recovery is associated with a remapping of the initial spatial input representation. A) In the model, the color code represents the spatial location of the input that the so-colored neuron was strongest responding to before the lesion (left column). The LPZ appears black early after the lesion (middle column), since neurons in the LPZ are deprived of any external input and horizontal connections are not strong enough to cause supra-threshold activation. Late after the lesion (right column), increased horizontal input to the LPZ contributed to an enlargement of representations from the peri-LPZ into the LPZ and thereby to a ‘filling’ of the LPZ with adjacent representations. Note the color gradients from top to bottom for all six columns in each of the three panels in A. Also compare Fig. S1. B) Retinotopic remapping in mice before lesion (left panel), at day 7 (middle panel) and at day 17 (right panel). (Reprinted by permission from Macmillan Publishers Ltd: Nature Neuroscience: Keck T et al. (2008) Massive restructuring of neuronal circuits during functional reorganization of adult visual cortex. Nat Neurosci 11:1162–1167, copyright 2008). In V1 of the mouse cortex, colors visualize the spatial position of the retinal input. Areas are colored according to which input they respond most strongly to. White dashed lines indicate the LPZ. Scale bar represents 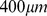.

**Figure 9:**
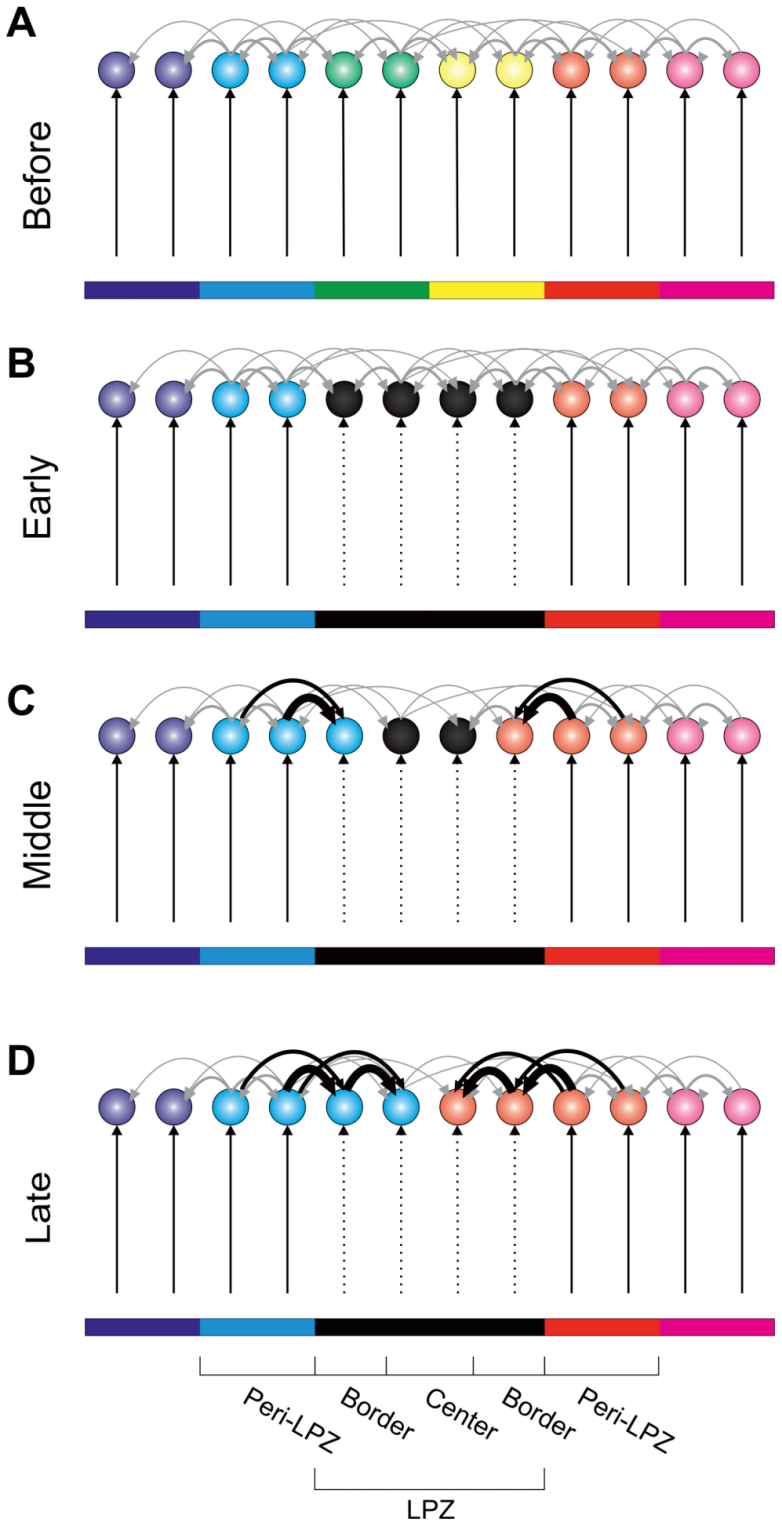
Schematic figure showing how structural plasticity in the model gives rise to functional network reorganization. New horizontal connections form between neurons in the peri-LPZ and the border of the LPZ as well as between the border and the center of the LPZ. In the model, these new synapses are the source for functional reorganization comparable to cortical retinotopic remapping. A) Network before lesion. Colors indicate spatial locations in an input layer like the retina (not explicitly modelled) and the strongest neuronal response in the model network or the primary visual cortex. Vertical input from the eye is in black, horizontal recurrent connections are in gray. Linewidth indicates the number of synapses. B) Black bar indicates loss of input. Vertical input to this area is permanently removed (dashed arrow lines). Silent neurons in the LPZ early after the lesion are labeled black. C) Additional synapses are formed at the rim of the LPZ (bold black arrows) in the middle phase after the lesion. Due to additional horizontal synapses, neurons in the border become active in response to adjacent input representation (blue and red). D) Late after the lesion, additional synapses from the border to the center contribute to the activation of center neurons, which now also become sensitive to adjacent representations (blue and red).

Many studies have shown that a loss of input due to a focal retinal lesion causes a remapping of retinotopic representations in the primary visual cortex [71]–[80]. Traditionally, cortical remapping is thought to be caused by changes in synaptic strengths, for instance due to STDP [48]. Recently it has been shown that, in the primary visual cortex, lesion-induced functional remapping coincides with massive structural changes in dendritic spines [10] and axonal branches and boutons [13]. To investigate whether retinotopic remapping could be accounted for by our *structural plasticity model*, we tested spatial input representations in the model at different time points before and after the lesion (Fig. 8a). This was done in line with experimental procedures to assess retinotopic mappings [10], as follows: The plane of neurons was subdivided into 6×6 non-overlapping rectangular areas. Subsequentially, each area received a noisy test current with mean 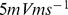 and standard deviation 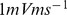 for 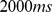. During the stimulation, we measured the number of action potentials in all neurons of the network in order to determine which areas of the network responded to focussed input. At the time of stimulation, we froze the connectivity in the network so that the test stimulus did not change the retinotopic mapping, to test how focussed input spreads throughout the network via horizontal connections. Afterwards, we color-coded each neuron according to the input area it strongest responded to. Immediately after the loss of input (no test inputs were given to the neurons in the LPZ), all neurons inside the LPZ are irresponsive to any inputs given to any neurons outside the LPZ. In the early phase after the lesion, neurons at the edge of the LPZ start to fire in response to input from adjacent areas in the peri-LPZ. In the middle phase, bordering representations begin to enlarge from the peri-LPZ into the LPZ and thereby ‘fill’ the LPZ from the border to the center. In the late phase, even neurons in the center of the LPZ become responsive again. The course of remapping from the border to the center coincides with the formation of additional horizontal connections (Fig. 9), in the early phase from the peri-LPZ to the border of the LPZ, and in the middle and late phases from the border to the center. In the late phase, representations of the peri-LPZ have almost completely ‘filled’ the entire LPZ. Only in the very center of the LPZ did some neurons remain irresponsive, depending on the size of the lesion and the width of the kernel function (Eq. 9). Importantly, in our model the remapping arises purely from structural changes, such as synapse formation and reorganization of ‘horizontal’ intra-cortical connections. We did not impose any form of synaptic plasticity such as LTP or STDP. Therefore, remapping in the model is not the result of a goal-directed associative process but emerges from the interactions between local activity-dependent changes in spine and bouton numbers. The course of remapping resembles the remapping in mice visual cortex (Fig. 8b).

### Aberrant network reorganization

**Figure 10:**
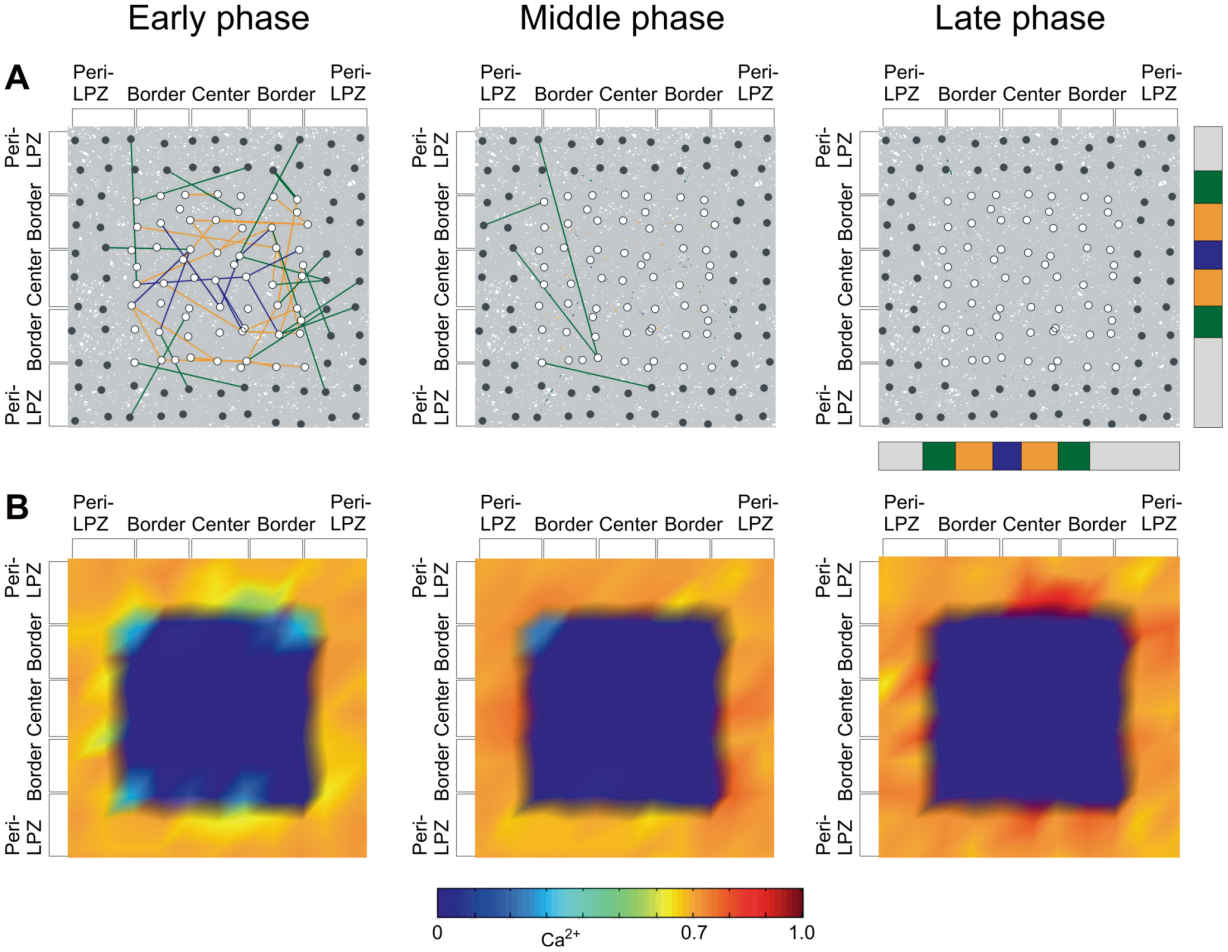
Network repair is not obtained if the minimum activity required for the formation of dendritic elements is higher than the activity inside the LPZ after the lesion. A) In this scenario with 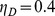, new synaptic connections impinging on neurons in the LPZ are formed only transiently in the early phase after the lesion when the activity of some neurons momentarily exceeds 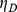. New connections are removed over time and even old connections are pruned. B) Activity in the LPZ decreases to a minimum level over time. Cf. Fig. 3 for color code.

So far we have shown that functional network reorganization similar to that observed experimentally can arise from local activity-dependent rules for axonal and dendritic element formation. Now we contrast these growth rules with different growth rules leading to aberrant network reorganization. Here we discuss two other cases with fundamentally different time courses of network reorganization. In the first aberrant case, the set-point 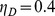 is relatively high compared to 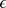 while 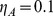 is rather low. In this scenario, network repair is impossible because neuronal activity in the LPZ does not exceed 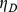 and therefore neurons reduce their dendritic elements instead of forming additional elements in a compensatory manner. Consequently, axonal elements from the peri-LPZ are not be able to find dendritic targets in the LPZ, so no new synapse are formed and neuronal activity cannot increase (Fig. 10). Thus, not only the high set-point 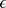 [35], [81] but also the minimum activities 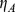 and 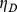 for axonal and dendritic element formation, respectively, are crucial for whether or not neurons can restore their activity level after the lesion.

**Figure 11:**
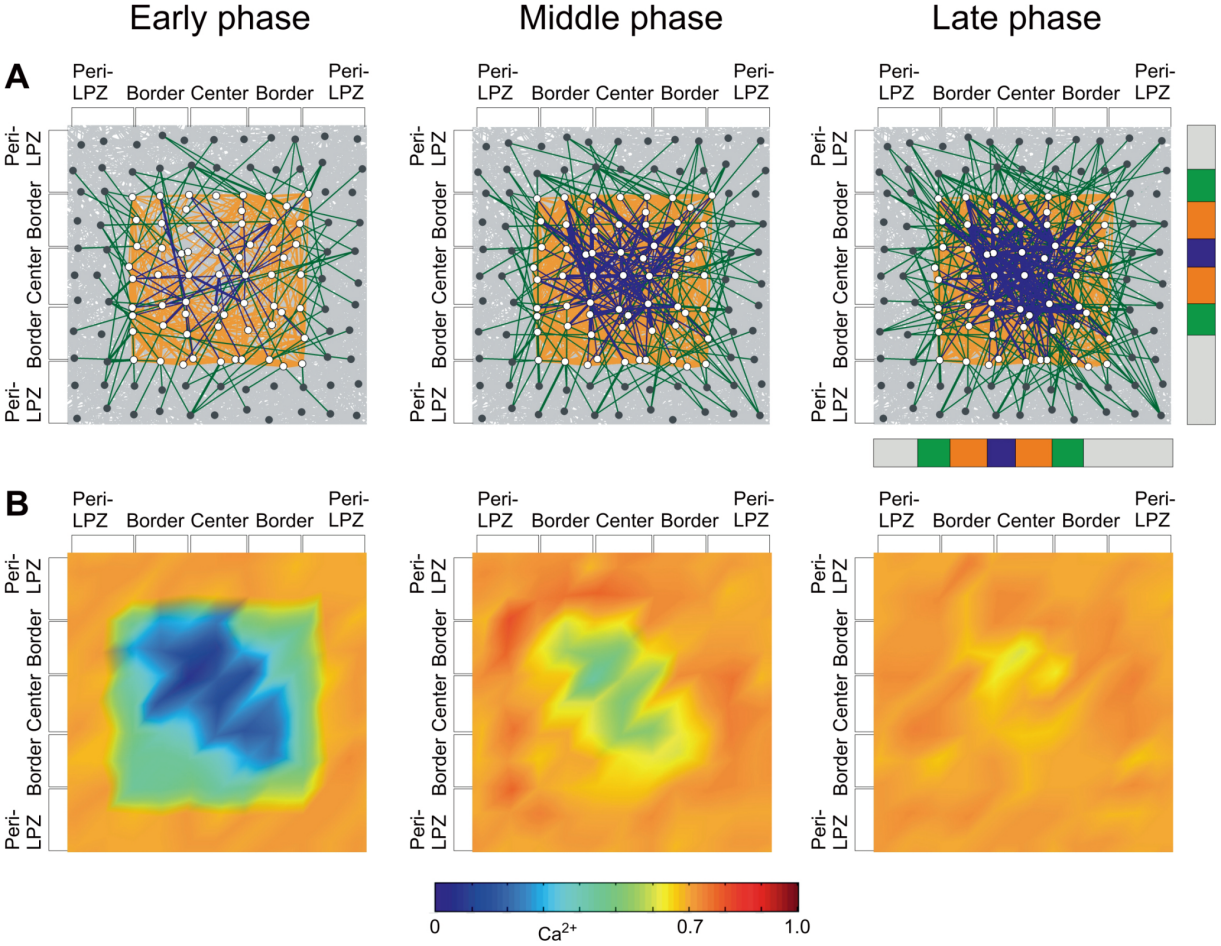
Different course of network repair for low 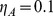 and 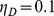. A) Network repair is mainly due to the massive formation of new recurrent connections in the border and the center of the LPZ, while ingrowing connections from the peri-LPZ into the LPZ are rare. Recurrent connections in the border are established first followed by recurrent connections from and within the center. The timing depends only on the initial activity levels rather than on cooperative effects between different areas. B) Initially, the activity is slightly higher in the border than in the center due to normal (non-deprived) activity spreading from the peri-LPZ into the border. Cf. Fig. 3 for color code.

**Figure 12:**
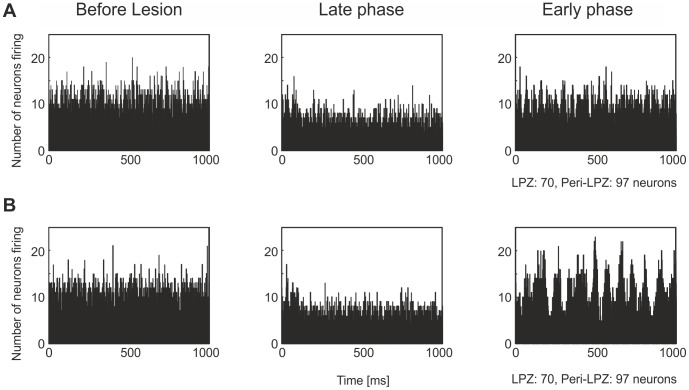
Electrical activity in terms of total number of firing neurons (excitatory and inhibitory) in time bins of 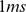 measured in the peri-LPZ and LPZ before and after the lesion. A) Physiological case of network reorganization, with 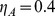 and 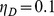. B) Aberrant case of network reorganization with 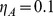 and 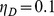. Before the lesion, the networks show irregular activity (left column). In the early phase after the lesion (middle column), the number of firing neurons is strongly reduced. In the late phase after the lesion (right column), the network either returns to a state of irregular activity (A), or turns into a state of more synchronized activity, with strong oscillations in electrical activity (B).

In the second aberrant case, 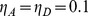 are equally low. In this scenario, neurons that are deprived of external input literally pull themselves up by their own bootstraps to restore their activity. Because the neurons simultaneously generate dendritic and axonal elements, massive recurrent connections are formed within the LPZ (Fig. 11). Recurrent connections enable the neurons to amplify fluctuations in activity and raise their average activity to the high set-point. Interestingly, the activity state of the network changes from irregular to more synchronized firing (Fig. 12), with clear oscillations in activity, when massive recurrent connections form. The time course of activity recovery differs fundamentally from the time course of normal network repair (cf. Fig. 3), in that neurons do not sequentially return to the high set-point. Neurons with the same initial activity level recover simultaneously.

**Figure 13:**
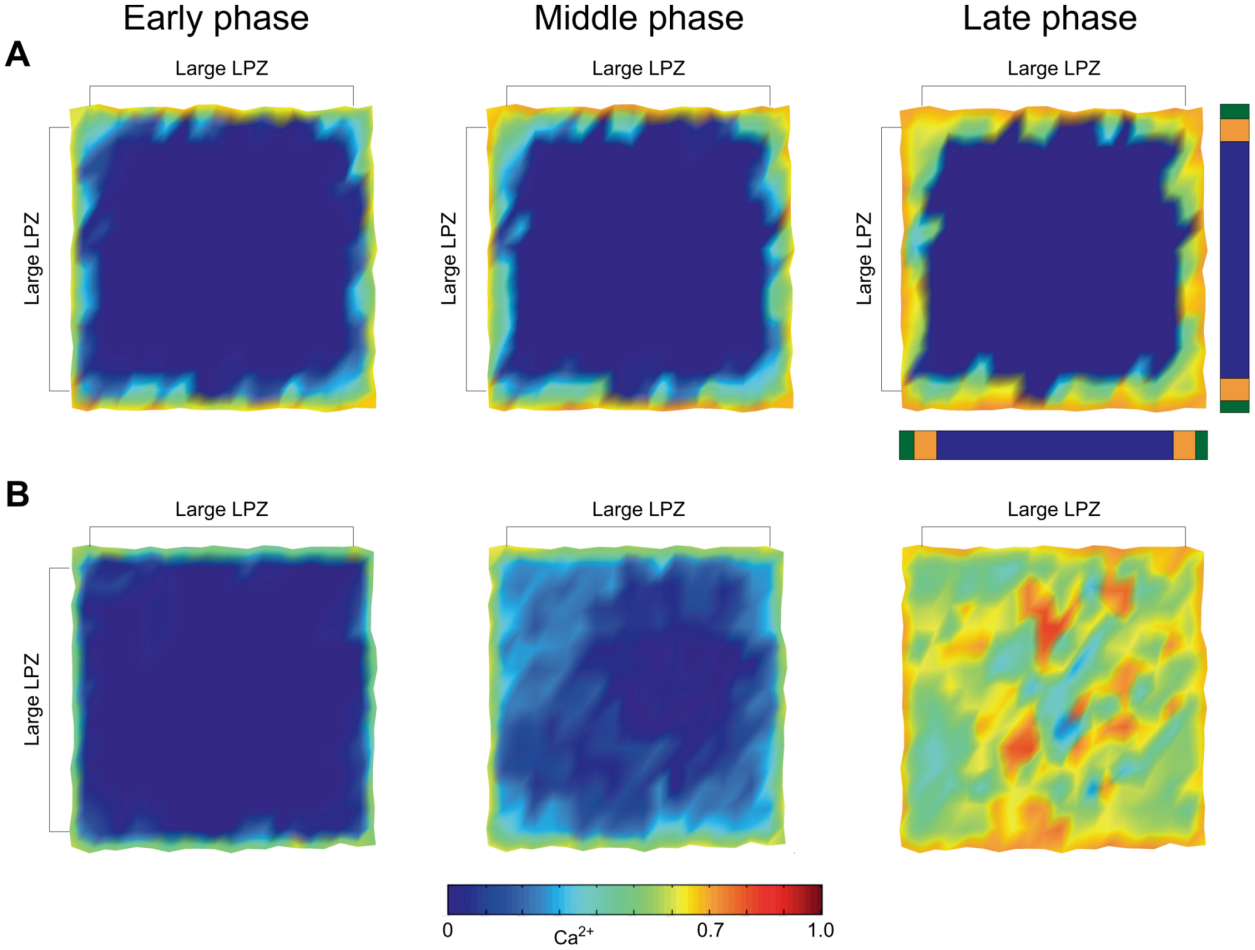
Whether neurons in large LPZs recover depends on the growth parameters 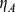 and 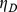. A) Network repair of large LPZs does not occur for low 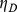 and high 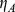, although some network reorganization takes place in the peri-LPZ and the border of the LPZ. B) Even in large LPZs neurons can completely recover if both 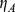 and 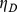 are low and the remaining activity in the LPZ allows axonal and dendritic elements to increase in number. Bars next to the top right panel indicate the size and position of the LPZ (orange and blue) and the peri-LPZ (green) relative to the entire network. Total number of neurons (excitatory and inhibitory): LPZ: 328 neurons; peri-LPZ: 72 neurons.

The difference between the different scenarios becomes especially clear for LPZs that cover almost the entire network. Networks with a high 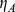 and a low 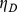 (physiological case) do not allow for network repair when (almost) the complete external input is removed (Fig. 13a) but only show some restricted reorganization in the border region of the LPZ. By contrast, networks with equally low 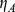 and 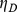 do recover well (Fig. 13b). In fact, LPZs of any size will recover as long as the remaining activity in the LPZ after the lesion exceeds 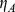 and 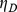. Large LPZs most clearly show the simultaneous recovery of neurons. Furthermore, due to the formation of massive recurrent connections, neurons frequently become hyperactive after network rewiring. This structural dynamics is comparable to that described in [35], [81].

**Figure 14:**
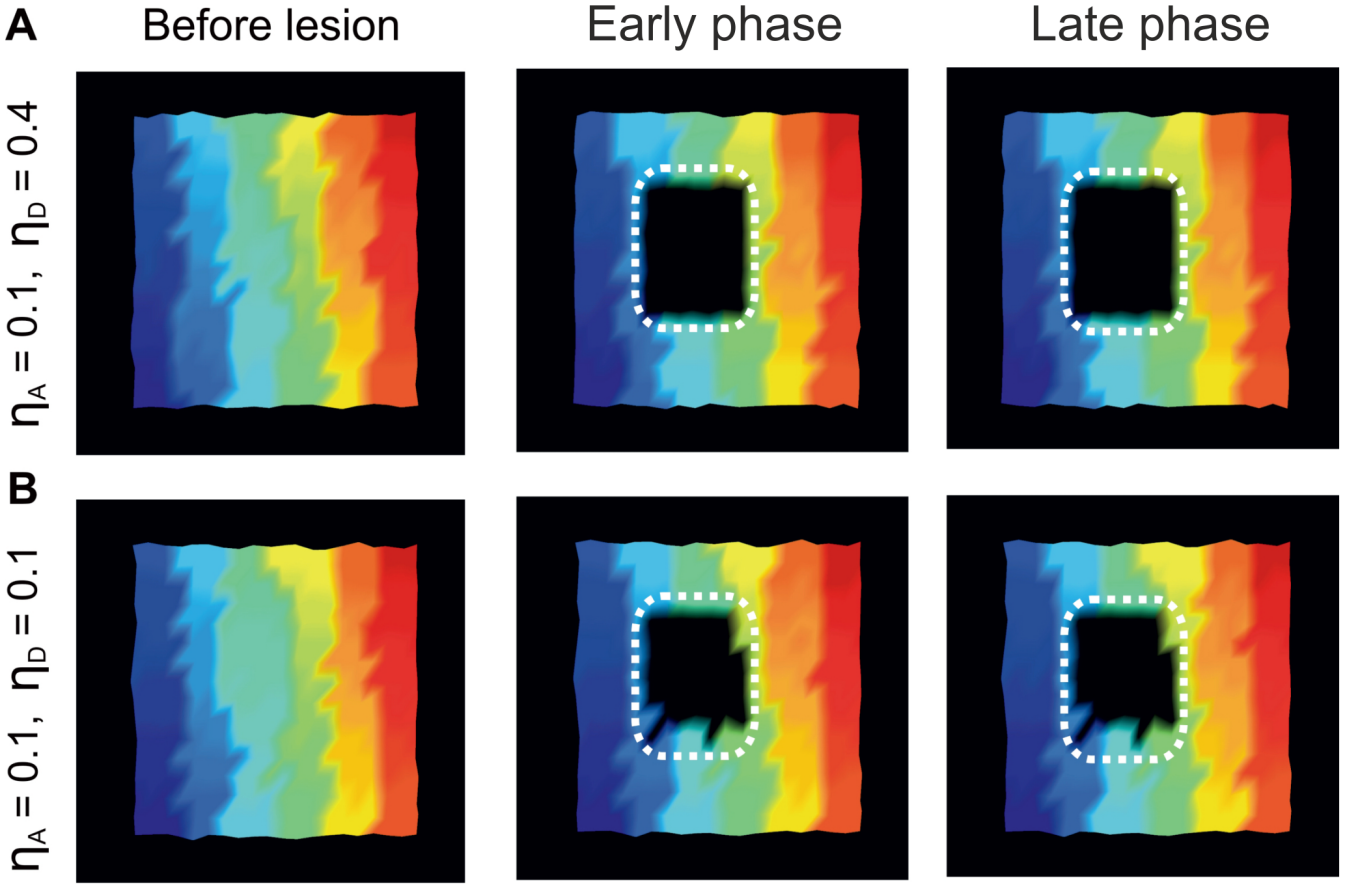
No functional network reorganization in terms of remapping of input representations occurs for the case with 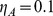, 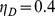 and the case with 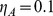, 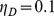. While the first case (A) is trivial because the neurons do not recover at all, the second case (B) is in so far remarkable that rewiring brings the activity back to the high set-point but does not give rise to functional remapping. Even in the late phase, the neurons in the LPZ, apart from a few neurons at the rim, do not become responsive to adjacent input. Cf. Fig. 8 for color code. Note the color gradients from top to the bottom for all six columns of the three panels in A and B.

As shown in Fig. 9, functional reorganization in terms of the remapping of spatial input representations is the result of a sequential formation of synapses between axonal elements in the peri-LPZ and dendritic elements in the border of the LPZ, and thereafter between axonal and dendritic elements in the border and the center, respectively. This can be interpreted as a sequential ingrowth of axons from the peri-LPZ into the border and from the border into the center. Remapping therefore emerges from cooperative effects between the peri-LPZ and the LPZ, in the sense that new synaptic connections transmit additional activity into the LPZ. New synaptic connections help neurons in the LPZ to restore their activity to the high set-point, thereby enlarging the input representations from the peri-LPZ into the LPZ—the remapping observed. Both scenarios with aberrant network reorganization discussed here do not show this ‘ingrowth’ of new synaptic connections into the LPZ. For the case with 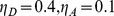, this is due to the lack of newly formed dendritic elements in the LPZ. As expected, functional reorganization in terms of remapping does not occur (Fig. 14a). Interestingly, remapping is also absent in the case with 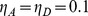 (Fig. 14b), in spite of robust network repair in the sense that all neurons returned to the high set-point. Network repair is in this case caused by recurrent synaptic connections within the LPZ and hardly by newly formed connections from the peri-LPZ into the LPZ. The lack of remapping in this scenario supports the notion that ingrowing connections are crucial for remapping. It further demonstrates that functional reorganization depends on growth rules with the right proportion of activity-dependent increase and decrease of axonal and dendritic elements. Thus, the values of 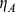, 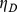 and 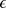 are crucial in determining the principally different types of network reorganization. Network parameters such as neuron numbers or neuron densities or neuronal parameters for electrical activity and calcium dynamics are not critical, and the same results can be obtained with other choices of these parameters (cf. Fig. S1).

### Predictions

We assumed biologically plausible growth rules inspired by what is known from in vitro studies about how neurons reshape their morphology in response to changes in electrical activity. The precise morphological response of neurons in the visual cortex to changes in activity has never been determined. Our modeling study revealed that growth rules adopted from in vitro studies can produce structural and functional network dynamics that show striking similarities to experimentally observed changes in the primary visual cortex after focal retinal lesion. Thus, we predict that activity-dependent changes in dendritic spines and axonal boutons of visual cortex neurons are governed by growth rules that purely depend on the cell's own level of activity. These growth rules can be described by simple Gaussian-shaped curves, and state that neurons have a narrow range of activity for which axonal and dendritic outgrowth, or at least axonal bouton and dendritic spine formation, is optimal, and that very high and very low activity cause retraction. It has never been considered in the context of retinotopic remapping that neurons may retract horizontal connections when their activity is too low. Furthermore, to obtain functional remapping, axonal and dendritic growth rules should not be identical but dendrites must start growing at lower activity levels than axons (Table 1).

**Table 1:** The outcome of network reorganization depends on the morphological response properties of individual neurons.

	Network repair	Network repair sequentially	Network repair of large PLZ	Functional Remapping
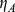 low, 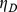 high	No	No	No	No
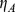 low, 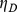 low	Yes	No	Yes	No
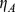 high, 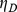 low	Yes	Yes	No	Yes
Experimental data	Yes	Yes	No	Yes
Kohonen model	Yes	No	Yes	Yes

The parametes 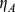 and 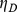 indicate the minimum average electrical activity individual neurons need to form axonal and dendritic elements, respectively. Experimental data is based on [10], . Kohonen model according to [89], [90]. Only for 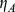 high, 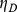 low, the model matches experimental data (cf. third and fourth row).

**Figure 15:**
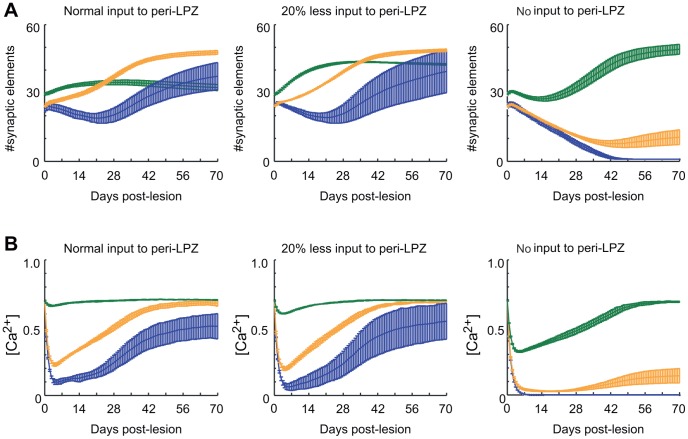
We predict that network repair is influenced by external electrical stimulation. During recovery without additional stimulation, the formation of dendritic elements in the border (orange) and center of the LPZ (blue) together with the activity-dependent formation of axonal elements in the peri-LPZ (green) contribute to an increase in connectivity between the peri-LPZ and LPZ and to the recovery of the network. This is revealed by the increasing numbers of synaptic elements on excitatory neurons (A, first panel) and by the change in calcium levels (B, first panel) in the border and center of the LPZ. A slight reduction in the external input to the peri-LPZ by 20% gives rise to an increase in the production of axonal elements on excitatory neurons (A, second panel) and an increase in the calcium concentration (B, second panel). A complete blockade of activity in the peri-LPZ, however, prevents recovery (B, third panel), because it suppresses the formation of axonal elements in the early phase of the recovery while dendritic elements in the LPZ start degenerating (A, third panel). Error bars: SD.

As a further consequence of the growth rules, we predict that the presence of different activity levels inside and outside the LPZ is what drives the rewiring of the network after the lesion. To support this prediction, we lowered the external input to the peri-LPZ in the model by either 20% or by a complete blockade. We could suppress compensatory network reorganization significantly by blocking external input to the peri-LPZ (Fig. 15, right column). This indicates that the recovery of the LPZ indeed crucially depends on the higher activity level in the peri-LPZ than in the LPZ. Interestingly, a slight reduction in the input to the peri-LPZ by 20% (Fig. 15, middle column) had a promoting effect on axonal element formation (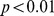 for data points later than post-lesion day 7; Mann-Whitney U test). This is because the axonal growth rule reaches a maximum for activities slightly below the set-point. Since the ‘filling’ of the LPZ with adjacent representations depends on the size of the lesion relative to the width of the kernel function (Eq. 9), we further predict that larger LPZs may functionally recover at the rim but will not completely reorganize. Since all elements of the growth rules, such as electrical activity, intracellular calcium and dendritic and axonal morphology, can be measured experimentally, our predictions are amenable to experimental testing.

Taken together, our work points to the need to quantify the activity-dependent growth of neurons in the visual and other cortices in order to understand better how alterations in sensory input cause the cortex to rewire its circuitry.

## Discussion

By means of our *structural plasticity model*, we have shown that specific, functional network reorganization can emerge from local homeostatic growth rules. The dynamic changes in axonal and dendritic elements produced by these growth rules were comparable to the dynamics of dendritic spine remodelling and axonal sprouting observed in the primary visual cortex after focal retinal lesions in mice [10] and in monkeys [13], respectively. We therefore conclude that neurons in V1 may have a narrow range of electrical activity in which axonal and dendritic growth is optimal. Importantly, axons may need higher activitity to grow out than dendrites. Initial activity differences, with higher activities in the peri-LPZ and lower activities in the border region of the LPZ, may drive axonal outgrowth in the peri-LPZ and dendritic spinogenesis in the border of the LPZ, respectively. New synaptic connections from the peri-LPZ to the border of the LPZ restore the activity from the outside to inside of the LPZ. Indeed, experimental studies have shown that a circular wave front of electrical activity travels slowly from the edge of the LPZ inwards [80]. Our model indicated that if the area of deprived input is not too large, cortical reorganization is capable of restoring electrical activity of most neurons in the LPZ. Interestingly, quantitative changes in the long-term neuronal activity markers c-fos and zif268 indeed suggest that neurons inside the LPZ strive towards homeostasis in electrical activity [82], [83]. Therefore, we conclude that homeostatic regulation may serve as a fundamental principle in reshaping cortical circuitry after focal loss of input.

The growth rules postulated in our model try to capture the essential aspects of activity-dependent axonal and dendritic changes reported in experimental studies. Dendritic spines need a certain level of electrical activity to persist [28], [30], which corresponds to our model assumption of a minimum activity level required to maintain dendritic elements (the first set-point 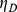). Incidentally, the minimum required level of activity measured experimentally is unexpectedly low [28], in line with our model result that 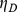 needs to be lower than 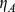 for functional reorganization. Reducing electrical activity, but not below the minimal required level [28], promotes the formation of spines [66], [84], [85], enabling neurons to explore the neuropil for axonal contacts [86]. Our model assumption that lowering electrical activity increases the neuron's number of dendritic elements is thus supported by experimental findings. Finally, increasing electrical activities attenuates dendritic growth, while overstimulation may lead to dendritic pruning [65]. This is reflected by the model assumption that too high activity is not beneficial for the formation of dendritic elements. Axonal growth, too, is modulated by electrical activity, and the way axons respond to activity is comparable to how dendrites respond. Blockade of activity can promote axonal sprouting and synapse formation, whereas increasing the activity level can suppress axonal outgrowth [17]. The precise levels of 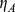, 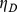 and the homeostatic set-point 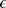 remain to be quantified experimentally.

The model showed that functional reorganization can arise from local structural plasticity alone and does not require associative forms of plasticity such as LTP or STDP. Sprouting of axonal elements from the peri-LPZ into the LPZ gives rise to an enlargement of representations adjacent to the LPZ and consequently to a remapping of spatial input representations in the network. Although the number of neurons in the network 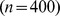 is relatively low compared to the number of neurons in the primary visual cortex, we believe that the essential dynamics in the model, with network repair and retinotopic remapping following focal retinal lesions [71]–[80], is not dependent on neuron number. We simulated a network with a higher neuron density (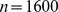 neurons in a network of the same physical size as before with 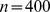 neurons) and obtained the same structural and functional network reorganization (Fig. S1).

Although axonal sprouting was found following focal retinal lesions [12], [13], [70], it has as yet not been possible, because of methodical limitations, to show that new axons establish synapses with deafferented neurons. On the basis of our modeling results, we postulate that the formation of additional horizontal connections from the peri-LPZ into the LPZ may account for the retinotopic remapping as seen, for example, in mice [10]. Since axonal growth in the mature cortex is restricted to a few hundreds of micrometers [54], this would further explain why the compensatory capacity of cortical networks is limited and larger LPZs do not recover [87], [88]. Nevertheless, as our *structural plasticity model* predicts, functional recovery at the rim of the LPZ usually takes place even with larger lesions [80].

Our model stands in contrast to the Kohonen model for self-organizing maps [89], [90], traditionally applied to explain cortical remapping. In the Kohonen model, cortical neurons receive input from all cells in the retinal input layer and merely change their response properties towards intact retinal input. However, there is strong experimental support that horizontal connections within the cortex in fact are the source of functional reorganization [91], [92]. Moreover, our model also better resembles the experimentally observed time course of the ‘filling’ of the LPZ with adjacent representations from the border to the center [80], [83]. By contrast, the Kohonen model would predict a simultaneous change in the response properties of all neurons in the entire LPZ (cf. Table 1).

Our conclusion that structural plasticity in the cortex after retinal lesions [9], [10], [12], [13], [70] may contribute to cortical remapping complements the current notion that retinotopic remapping could arise from synaptic plasticity [93], in particular from spike timing-dependent plasticity [44], [48]. Our modeling results are the first to indicate that cortical remapping may not necessarily require associative plasticity but can already emerge from structural reorganization of synapses (structural plasticity) alone. In the living brain, cortical remapping is most likely the result of both structural and synaptic plasticity, but the power of a modelling approach is that the impact of both types of plasticity can be explored separately. In our future models, we intend to include both types of plasticity and investigate whether synaptic plasticity can contribute to the experience-dependent (fine) tuning of structurally formed cell assemblies [9], [94] and the experience-dependent formation of memory traces therein.

An important question is how inhibitory neurons contribute to network repair. A recent study [95] has shown that inhibitory neurons respond to a loss of input with a rapid decrease in structural connectivity that is strongest in the center of the LPZ and fades with distance to the LPZ. It needs to be tested how the dramatic loss of inhibition influences the activity in the network. A straightforward interpretation is that disinhibition of neurons contributes to the difference in activity between the LPZ and peri-LPZ. Waves of spreading activity are a common phenomenon after brain lesions, for example after focal stroke [96]. Modeling work has revealed complex interactions between activity and structure, with various forms of oscillatory activity, when inhibition was substantially altered [81].

Previous work on cortical reorganization after loss of visual input has raised the question of whether synapse formation is specific or unspecific [9]. The observation that synapse formation takes place particularly at the edge between areas with intact and deprived input led to the notion that cortical organization may be highly specific [9], [80]. Associative forms of synaptic plasticity such as STDP [48] may serve as such specific mechanisms. However, these mechanisms require that neurons keep track of their current firing and, in case of STDP, even of the firing history of other neurons. Here we propose an unspecific mechanism that can give rise to specific cortical reorganization. We dub this mechanism *neuron-centric* plasticity, since neurons change their morphology purely on the basis of their own level of electrical activity, i.e. form synaptic elements and offer them to the neuropil [49], [50]. Remarkably, without knowing the activity levels of other neurons and randomly recombining their vacant synaptic elements, neurons can achieve homeostasis in electrical activity (at both the cellular and the network level) and even cause functional cortical remapping. This notion stands in sharp contrast to associative, synapse-centric forms of plasticity such as STDP, in which the timing relations between pre- and postsynaptic activity determine the strengthening or weakening of synapses, and which are traditionally regarded as the standard paradigm for cortical reorganization.

Other forms of neuron-centric plasticity, such as synaptic scaling, also contribute to homeostasis in electrical activity [97]. Neurons in the cortex [98], hippocampus [99] and spinal cord [100] can up- or down-regulate all their incoming synapses when their firing rates become too low or too high, respectively. The main difference with network reorganization by activity-dependent structural plasticity is that synaptic scaling has an immediate effect on postsynaptic firing rates, whereas activity-dependent structural plasticity requires the availability of complementary synaptic elements for synapse formation. Synaptic scaling and structural plasticity operate on different time scales, but may also cooperate. Neurons may form synapses de novo to explore new sources of activity when synaptic scaling has become insufficient to maintain activity homeostasis, for example because presynaptic neurons are also inactive.

We propose that activity-dependent structural plasticity may provide a unifying framework for understanding cortical reorganization in a wide range of situations including network repair in degenerative diseases or following focal stroke.

## Supporting Information

Figure S1Structural and functional recovery does not depend on the density of neurons. Here we used 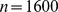 neurons of which 1280 are excitatory and 320 are inhibitory with an expected distance between the excitatory neurons of 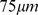 along the x,y-coordinates (giving the same network size as in simulations with 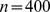 neurons). Inhibitory neurons are placed between the excitatory ones as described in “Neuron model for electrical activity”. A) All neurons used have reached the high set-point 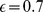 before lesion onset (left panel). In the early phase after the lesion, calcium concentrations are low in the entire LPZ, with the lowest calcium concentrations in the center (middle panel). Most of the neurons in the LPZ are able to return to the high set-point in the late phase of the lesion (right panel). Every white dot indicates the position of an excitatory neuron. B) Even in networks with high neuron densities, structural network repair goes along with cortical remapping. Every color indicates the localization of the spatial input that each neuron was strongest responding to. Remapping was assessed as in Fig. 8A. Note the color gradients from top to the bottom for all six columns of the three panels in B.(TIF)
