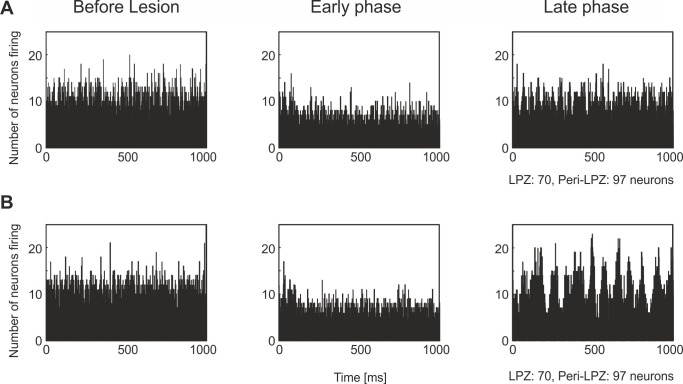# Correction: A Simple Rule for Dendritic Spine and Axonal Bouton Formation Can Account for Cortical Reorganization after Focal Retinal Lesions

**DOI:** 10.1371/annotation/e8b7df48-4639-4ac1-8a98-cb13dea3415b

**Published:** 2013-10-23

**Authors:** Markus Butz, Arjen van Ooyen

There was an error in the headings of Figure 8. Please see the corrected figure here: 

**Figure:**
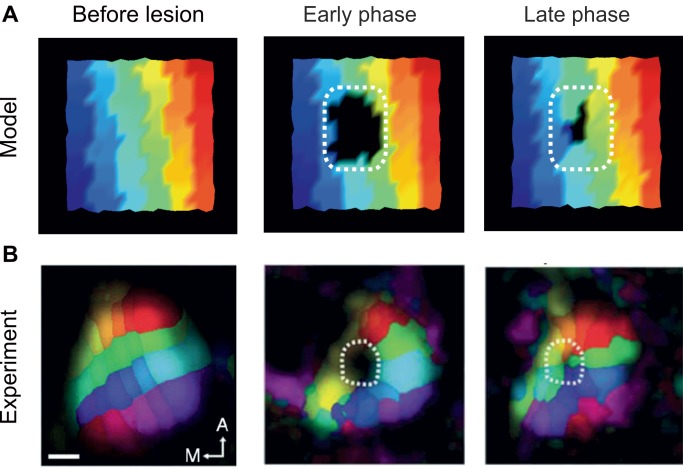


There was also an error in the headings of Figure 12. Please see the corrected figure here: 

**Figure:**