# Supplementary figures and images for: A Simple Rule for Dendritic Spine and Axonal Bouton Formation Can Account for Cortical Reorganization after Focal Retinal Lesions
Source: PLoS Comput Biol. 2013 Oct 10;9(10):e1003259. doi: 10.1371/journal.pcbi.1003259 (PMC3794906; doi:10.1371/journal.pcbi.1003259)

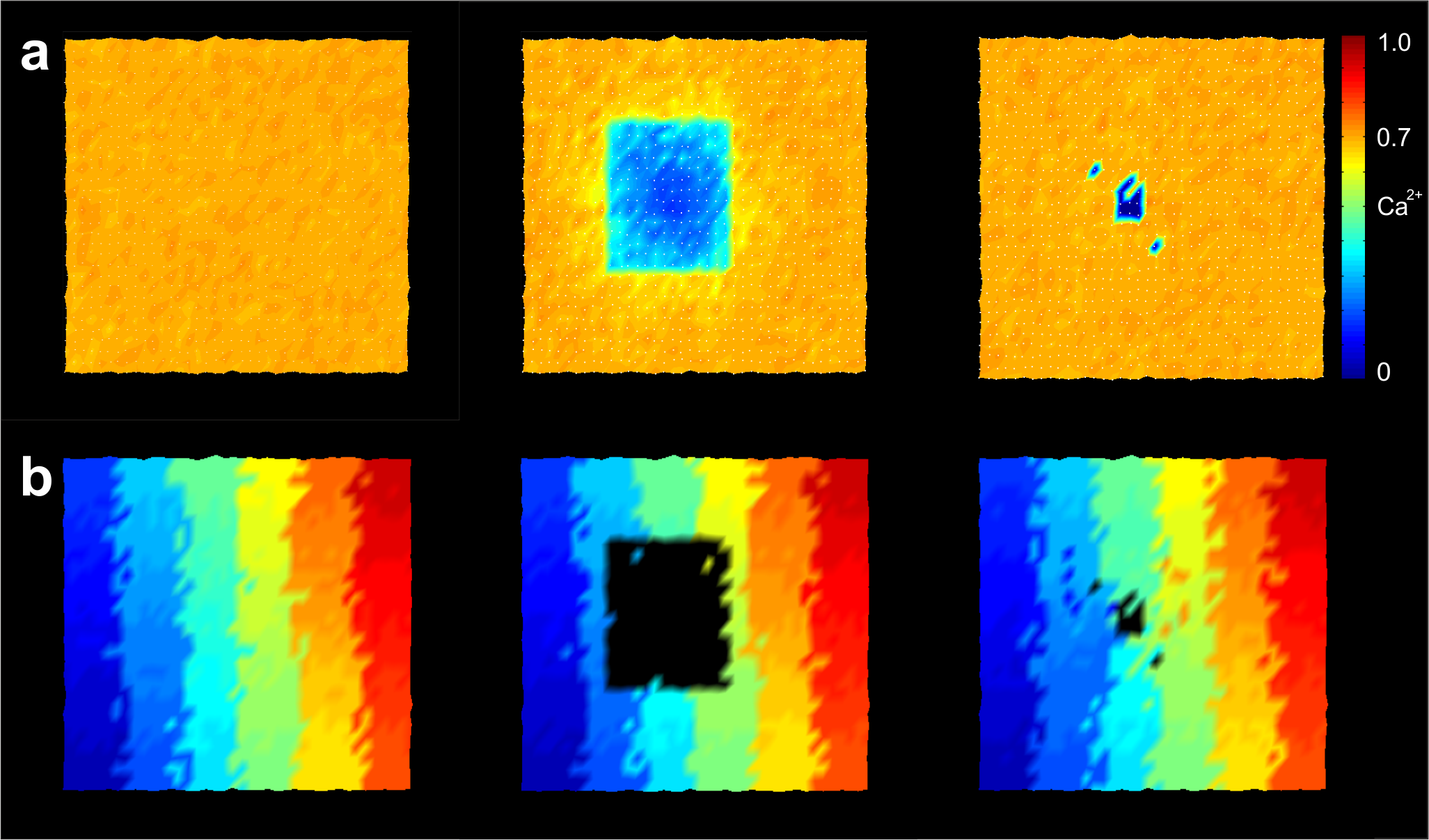

Supplement: Figure S1 — Structural and functional recovery does not depend on the density of neurons. Here we used neurons of which 1280 are excitatory and 320 are inhibitory with an expected distance between the excitatory neurons of along the x,y-coordinates (giving the same network size as in simulations with neurons). Inhibitory neurons are placed between the excitatory ones as described in “Neuron model for electrical activity”. A) All neurons used have reached the high set-point before lesion onset (left panel). In the early phase after the lesion, calcium concentrations are low in the entire LPZ, with the lowest calcium concentrations in the center (middle panel). Most of the neurons in the LPZ are able to return to the high set-point in the late phase of the lesion (right panel). Every white dot indicates the position of an excitatory neuron. B) Even in networks with high neuron densities, structural network repair goes along with cortical remapping. Every color indicates the localization of the spatial input that each neuron was strongest responding to. Remapping was assessed as in Fig. 8A. Note the color gradients from top to the bottom for all six columns of the three panels in B. (TIF) [file pcbi.1003259.s001.tif]
